# Intrinsic and regulated properties of minimally edited trypanosome mRNAs

**DOI:** 10.1093/nar/gkz012

**Published:** 2019-01-30

**Authors:** Brianna L Tylec, Rachel M Simpson, Laura E Kirby, Runpu Chen, Yijun Sun, Donna J Koslowsky, Laurie K Read

**Affiliations:** 1 Department of Microbiology and Immunology, University at Buffalo Jacobs School of Medicine and Biomedical Sciences, Buffalo, NY 14203; 2 Department of Microbiology and Molecular Genetics, Michigan State University, East Lansing, MI 48824; 3 Department of Computer Science and Engineering, University at Buffalo, Buffalo, NY 14260

## Abstract

Most mitochondrial mRNAs in kinetoplastids require extensive uridine insertion/deletion editing to generate translatable open reading frames. Editing is specified by *trans*-acting gRNAs and involves a complex machinery including basal and accessory factors. Here, we utilize high-throughput sequencing to analyze editing progression in two minimally edited mRNAs that provide a simplified system due their requiring only two gRNAs each for complete editing. We show that CYb and MURF2 mRNAs exhibit barriers to editing progression that differ from those previously identified for pan-edited mRNAs, primarily at initial gRNA usage and gRNA exchange. We demonstrate that mis-edited junctions arise through multiple pathways including mis-alignment of cognate gRNA, incorrect and sometimes promiscuous gRNA utilization and inefficient gRNA anchoring. We then examined the roles of accessory factors RBP16 and MRP1/2 in maintaining edited CYb and MURF2 populations. RBP16 is essential for initiation of CYb and MURF2 editing, as well as MURF2 editing progression. In contrast, MRP1/2 stabilizes both edited mRNA populations, while further promoting progression of MURF2 mRNA editing. We also analyzed the effects of RNA Editing Substrate Binding Complex components, TbRGG2 and GAP1, and show that both proteins modestly impact progression of editing on minimally edited mRNAs, suggesting a novel function for GAP1.

## INTRODUCTION

*Trypanosoma brucei* belongs to an early branching class of eukaryotes termed Kinetoplastea, several members of which are the causative agents of parasitic diseases in humans and livestock (1–3). Kinetoplastids are named after their unique mitochondrial DNA consisting of dozens of concatenated ∼22 kb maxicircles and several thousand ∼1 kb minicircles (4). Maxicircles contain 18 protein coding genes, 12 of which contain only very short open reading frames that lack homology to known mitochondrial proteins and are not believed to be translated into proteins. The mRNAs transcribed from these 12 genes require uridine (U) insertion/deletion editing to generate translatable mRNAs (5). Of the 12 edited mRNAs, 9 require modification throughout their lengths and are termed pan-edited and 3 mRNAs are edited over a much smaller region and are called minimally edited. Minicircles contain the genes for guide RNAs (gRNAs), non-coding RNAs which direct the insertion and deletion of Us from mRNAs. This process is accomplished in concert with several multiprotein subcomplexes including the RNA Editing Substrate Binding Complex (RESC; aka, MRB1 complex), the enzymatic RNA Editing Core Complex (RECC; aka, 20S editosome), and other editing accessory factors (6,7).

We previously employed high-throughput sequencing of edited mRNAs and subsequent analysis using the Trypanosome RNA Editing Alignment Tool (TREAT) developed in our laboratory to investigate editing progression in both wild-type cells and those depleted of specific editing factors (8–10). This method gives us quantitative nucleotide-level resolution of partially edited transcripts and is more powerful in assessing editing states than previously used methods. Using TREAT, we showed that editing of pan-edited mRNAs RPS12 and ND7-5′ is characterized by the presence of Intrinsic Pause Sites (IPSs; Table 1) interspersed throughout gRNA-defined blocks, pointing to natural barriers in the utilization of gRNAs. In addition, we showed that knockdowns of specific RESC factors produced Exacerbated Pause Sites (EPSs; Table 1) in edited transcripts that provide insight into functions such as gRNA utilization (as with TbRGG2 and MRB8180) or gRNA exchange (as with GAP1/2).

**Table 1.** Glossary of terms

Term	Definition
**Editing Site (ES)**	Any space between two non-T nucleotides (cDNA) has the potential to be edited at the RNA level and is termed an Editing Site (ES). ESs are numbered from 3′ to 5′ following the direction of editing.
**Editing Stop Site**	Moving 3′ to 5′, the Editing Stop Site is the final (5′ most) ES that matches the canonical fully edited sequence correctly. All ESs 3′ of the Editing Stop Site match the canonical fully edited sequence.
**Intrinsic Pause Site (IPS)**	An Editing Stop Site at which the total number of sequences sharing this Editing Stop Site is greater than the outlier threshold. IPSs represent ESs at which canonical editing frequently pauses.
**Exacerbated Pause Site (EPS)**	Editing Stop Sites that significantly increase (*P* < 0.05, *q* < 0.05) upon depletion of a given protein are termed EPSs.
**Junction Start Site (JSS)**	The first ES, moving 3′ to 5′ that does not match the canonical fully edited sequence correctly (can match pre-edited or mis-edited).
**Junction End Site (JES)**	The 5′ most ES with any editing action, whether canonical or mis-edited.
**Junction Length (JL)**	The number of ESs contained within a junction, i.e. between the JSS and JES (e.g. a junction arising after Editing Stop Site 15 with a JES at ES20 would have a junction length of 5).

In contrast to the pan-edited RPS12 and ND7-5′ mRNAs, which require several gRNAs to complete their editing, the minimally edited CYb and MURF2 mRNAs require only two gRNAs each (11). Pan-edited and minimally edited mRNAs have differing RESC dependencies. TbRGG2, a central component of the RNA Editing Mediator Complex (REMC; a subcomplex of the RESC), impacts the 3′ to 5′ progression of editing along pan-edited mRNAs (9,12), with quantitative reverse transcriptase-polymerase chain reaction (qRT-PCR) studies showing little to no impact on minimally edited mRNAs (13,14). On the other hand, GAP1/2, a heterotetrameric complex that is essential for gRNA stability, affects the editing of most edited transcripts with the exception of COII which contains its own *cis*-acting gRNA and does not rely on GAP1/2-stabilized gRNAs (15,16). In addition, some editing accessory factors that are not stably associated with RESC have relatively specific effects on the editing of minimally edited CYb and MURF2 mRNAs. Two accessory factors addressed in this study are RBP16 and MRP1/2. RBP16 was identified as a Y-box protein with gRNA/pre-mRNA annealing and unwinding activity and whose depletion resulted in reduced levels of fully edited CYb mRNA and a destabilization of never-edited mRNAs, with little to no effect on the editing of pan-edited mRNAs (17–19). The heterotetrameric MRP1/2 complex similarly has RNA binding activity and promotes mRNA-gRNA annealing, and its depletion caused a reduction in fully edited CYb and RPS12 transcripts as well as destabilization of never edited mRNAs and marginal effects on other mRNAs (19–23). The aforementioned studies quantified fully edited and pre-edited mRNAs by poisoned primer extension or qRT-PCR. These methods have limitations involving examination of a very limited region of an mRNA and primer placement, respectively, which could mask some editing defects caused by depletion of specific proteins. Recent *in vivo* cross-linking studies support a role for MRP1/2 in editing of minimally edited mRNAs as they demonstrated a specific correlation between the binding of MRP1 and RESC component MRB8170 across minimally edited mRNAs (24).

Here, we use high-throughput sequencing and TREAT analysis to examine both initiation and progression of editing in the minimally edited mRNAs, CYb and MURF2. In wild-type cells, we observed efficient utilization of gRNAs relative to what was observed in pan-edited mRNAs. We also find evidence for junction formation via multiple mechanisms including incorrect gRNA utilization, misalignment of the canonical gRNA and inefficient gRNA anchoring. Our data also reveal promiscuous gRNA usage, as one incorrect gRNA apparently used to edit MURF2 was previously identified to guide editing of another mRNA. In knockdown studies, we show that RBP16 impacts primarily initiation of CYb mRNA editing, but plays a role in both initiation and progression of MURF2 editing. We also demonstrate a role for MRP1/2 in stabilization of fully and partially edited CYb and MURF2 mRNAs, with an additional role in MURF2 editing progression. We present evidence that RESC components, TbRGG2 and GAP1, modestly affect editing progression in both minimally edited mRNAs. Overall, our data demonstrate that while minimally edited mRNAs are in some ways edited more efficiently than pan-edited mRNAs, their editing remains a complex and error-prone process requiring a constellation of protein factors.

## MATERIALS AND METHODS

### Cell culture

Procyclic form (PF) *T. brucei brucei* 29-13 cells were grown in standard medium supplemented with 10% fetal bovine serum as described (17). Cells were also grown in the presence of 15 μg/ml G418 and 50 μg/ml hygromycin. The following RNAi cell lines used in this study, all derived from procyclic 29-13 cells, were previously published: GAP1 (16), TbRGG2 (25), RBP16 (17) and MRP1/2 (19). RNAi cell lines were grown as described with the addition of 2.5 μg/ml phleomycin.

### qRT-PCR

All RNAi cell lines were grown either uninduced or induced with the presence of 2.5 μg tetracycline for 3 days. RNA was harvested using Trizol (Invitrogen) and phenol:chloroform extraction followed by DNase treatment (DNA-free DNase kit; Ambion). Purity and intactness of the RNA was confirmed using a NanoDrop 1000 (Thermo Fisher Scientific) and agarose gel electrophoresis. cDNA was then created using 1.2 μg RNA and random hexamer primers and the Taq-man Reverse Transcriptase Kit. Primers (Integrated DNA Technologies) used to detect the levels of total CYb and MURF2 mRNAs and mRNAs encoding target proteins, along with amplicon lengths, are shown in Supplementary Table S1. Amplification was performed using a CFX Connect Real Time System (BioRad), and data were analyzed using BioRad CFX Manager 3.1. Two biological replicates with three technical replicates of each were used to determine the level of knockdown and total mRNA levels, and these were normalized to 18S rRNA and β-tubulin mRNA using the standard curve method. A threshold of no more than 40% mRNA level remaining when normalized to both standards in the induced cells was considered acceptable for deep sequencing.

### Western blot analysis

RNAi cell lines were grown either uninduced or induced with the presence of 2.5 μg tetracycline for 3 days. Protein samples were then prepared by resuspending 5 × 10^7^ cells in 50 μl sodium dodecyl sulphate-polyacrylamide gelelectrophoresis sample buffer (final concentration of 1 × 10^6^ cells/ml) and heating at 95°C for 15 min. Proteins were separated on a 12.5% polyacrylamide gel and then transferred to a nitrocellulose membrane. Membranes were blocked with TBST-milk for 1 h and probed with polyclonal antibodies against TbRGG2 (13), GAP1 (26), RBP16 (27) and MRP2 (27), followed by secondary antibody. Hsp70 was blotted as a loading control (antibodies were a gift from Jay Bangs, University at Buffalo). Blots were imaged using a ChemiDoc MP (BioRad).

### Sequencing sample preparation

*Trypanosoma brucei brucei* PF 29-13 cells were grown to mid-log phase and harvested using TriZol. RNAi cell lines were grown either uninduced or induced with 2.5 μg/ml tetracycline for 3 days and harvested with the same method. Total RNA was isolated using phenol:chloroform extraction followed by DNase treatment. RNA was tested for purity via NanoDrop 1000 (Fisher Scientific) and for ribosomal RNA intactness on a 1.2% TBE agarose gel. Gene-specific cDNA was created from 1.2 μg RNA using Superscript III Reverse Transcriptase and the 3′ primer specific to the gene being analyzed, which is complementary to never edited sequence just downstream of the edited region. cDNA was then amplified using the same 3′ primer and a 5′ primer complementary to never edited sequence just 5′ of edited region (Supplementary Table S1). To ensure that no sub-populations of cDNA were disproportionately amplified in the amplicon library generation, the linear range of the PCR reaction for each sample was determined using 1.2 μM primers and 2.5 μl of cDNA in a 25 μl test PCR reaction. To generate the amplicons used for MiSeq sequencing, samples were PCR amplified up to the cycle number corresponding to the center of the linear range for each sample as determined in the test PCR reaction. PCR products were then purified using the Illustria GFX PCR DNA Purification kit and eluted into 10 μl 10mM Tris–HCl, pH 8. Library preparation and paired-end Illumina MiSeq was performed as described previously (8,9) and below. Five biological replicates were performed for 29-13 cells and two biological replicates each were performed for uninduced and induced RNAi cell lines. The number of total and unique sequences obtained in each sample is shown in Supplementary Table S2. The sequencing data used in this study has been deposited in the Sequence Read Archive under accession number SRP131678.

### Library construction and sequencing

cDNA resulting from PCR reactions described above was quantified using the Picogreen Assay (Invitrogen), and the sizes of products were confirmed using the Advance Analytical Fragment Analyzer using standard sense reagents. A 50 μl index PCR reaction was carried out to attach dual indices and Illumina sequencing adapters. Twenty-five microliter of 2× KAPA HiFi HotStart Ready mix was combined with 5 μl Nextera XT Index primer 1 (N7xx) and Index primer 2 (S5xx) and added to 2 ng of cDNA for the PCR reaction. AMPure XP beads (Beckman Coulter Genomics) were used to purify the final libraries. Libraries were then quantified using the Picogreen assay and Library Quantification kit (Kapa Biosystems). The Advanced Analytical Fragment Analyzer was used to analyze the sizes of the cDNA libraries and confirm the presence of appropriate size ranges for both the edited and unedited transcript sizes as well as ligated sequencing adapters. The libraries were quantified using a Qubit fluorimeter (Thermo Fisher Scientific) and normalized to 10 nM based on the concentration and amplicon fragment size. The libraries were pooled and sequenced using Illumina MiSeq 300 cycle Paired End sequencing.

### Pre-processing of RNA-seq paired end reads

Paired end sequencing reads from the Illumina MiSeq were obtained in FASTQ format. The FASTQ files were merged using PEAR (Paired-End reAd mergeR) (28). The resulting reads were then merged using the FASTX-Toolkit (http://hannonlab.cshl.edu/fastx_toolkit/index.html) utility program fastq-collapser, which collapses identical sequences into a single sequence while maintaining read counts.

### Sequence alignment with TREAT

TREAT is a multiple sequence alignment and visualization tool consisting of a command-line alignment algorithm and a web-based interface for searching, viewing, and analyzing sequence results. TREAT is written in Go and is freely available under the GPLv3 license at http://github.com/ubccr/treat. TREAT v0.03 (9) was used in this study. To ensure that sequencing libraries represent comparable sequence populations and that a subset of transcripts was not over-amplified, the number of sequences was compared across samples (Supplementary Table S2). We compared total sequences (Supplementary Table S2A, C, E and G) and unique sequences derived from this total (Supplementary Table S2B, D, F and H). Within these populations, we further determine the number of Standard Alignments (those sequences with no non-T mismatches) and non-Standard Alignments (those sequences having non-T mismatches). We ensured that all samples being compared have similar numbers of sequences (typically less than two-fold difference) and that the majority population lacks non-T mismatches as these are excluded from our subsequent analysis (Supplementary Table S2). For final analysis of sequencing results, total sequences in each sample with no non-T mismatches (Standard Alignments) were normalized to 100 000 sequences, thereby allowing comparison of the relative abundance of sequences (8).

The numbers of pre-edited and fully edited transcripts are identified by TREAT based on an exact match to user-supplied pre-edited and fully edited template sequences. Knockdowns were tested for significant changes to pre-edited or fully edited transcript levels by calculating the average number of pre-edited or fully edited transcripts compared to the average of the eight uninduced samples used in this study. Significance of changes to pre-edited, partially edited, or fully edited transcript levels upon RNAi was determined using the Student's *t*-test. IPSs were determined as described previously (8). The threshold above which an editing site is considered an IPS is determined using the formula: Outlier threshold = (1.5 * IQR) + 3Q, excluding pre-edited and fully edited transcripts (29). Determination of EPSs was described previously (9). In some cases, we extracted only partially edited sequences and renormalized these to 100 000 sequences to better compare partially edited populations between uninduced and induced samples of a given RNAi line. Exact values for the renormalized counts of sequences sharing each Editing Stop Site in both replicates and the associated *P-* and *q*-values are shown in Supplementary Tables S3 and 4. Searches of the gRNA database constructed by Koslowsky *et al.* were performed as previously described (11). For analysis of junction sequences, the total number of sequences with a given junction length (0, 1–2, 11–13) was averaged across replicates and plotted using R for each knockdown and for all uninduced samples.

## RESULTS

### Initial characterization of major CYb and MURF2 mRNA populations and IPSs

**Figure 1.**
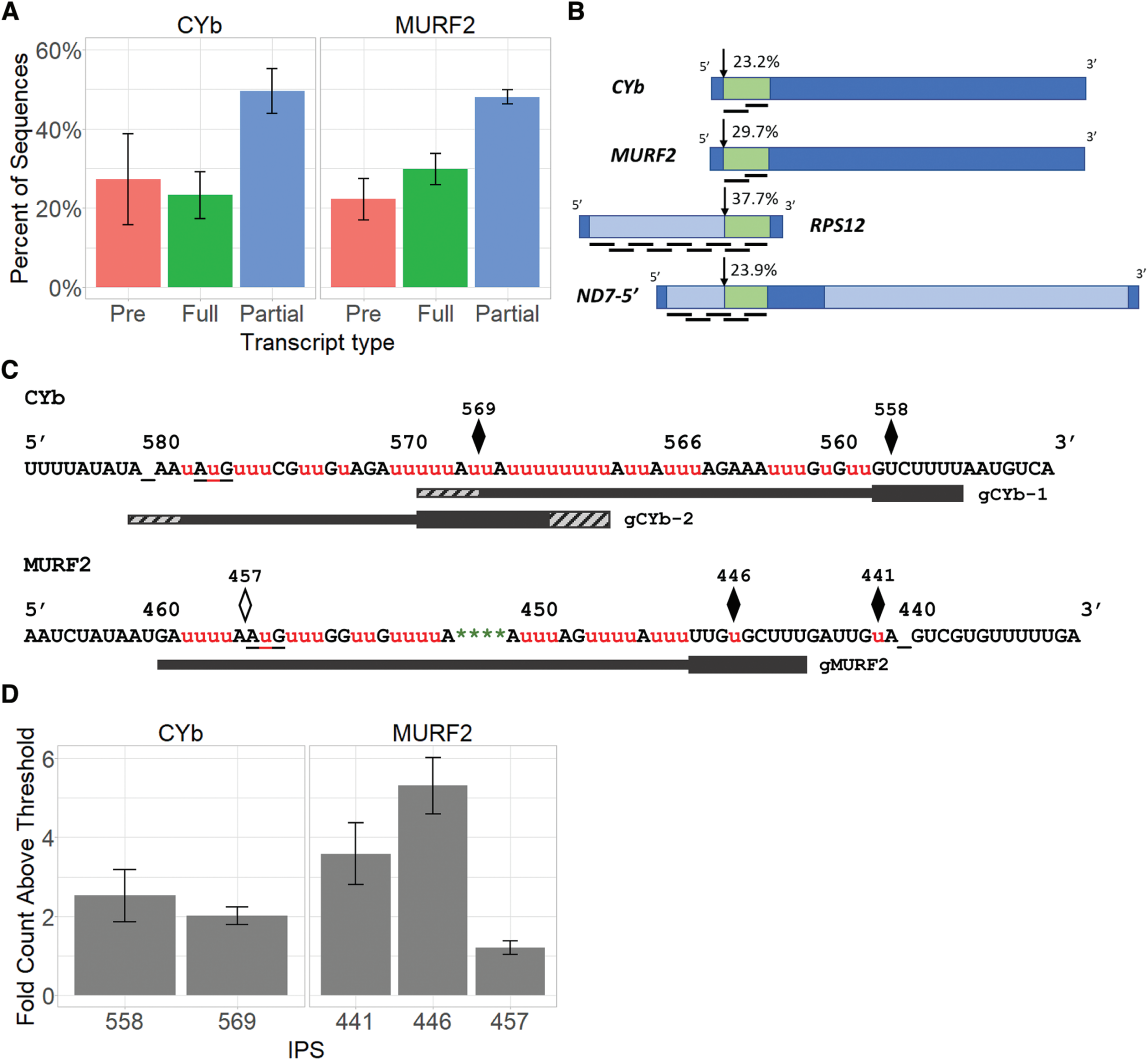
Sequence classes and IPSs in CYb and MURF2 editing. (**A**) The percentage of normalized pre-edited (Pre), fully edited (Full) and partially edited (Partial) sequences from *n* = 5 replicates are shown. Error bars represent one standard deviation. (**B**) Schematic (not to scale) of the mRNAs CYb, MURF2, RPS12 and ND7-5′ with associated gRNAs. The percentage of sequences containing fully edited sequence up to the end of the second gRNA are indicated. RPS12 and ND7 data are from (8). Dark blue, never edited region; light blue, region requiring further editing; green, region of fully edited sequence. (**C**) Locations of IPSs in CYb and MURF2 relative to edited mRNA sequence and known gRNAs. The sequences of the 5′ edited regions of both CYb and MURF2 are shown. Bars below the sequence represent gRNAs as published by Koslowsky *et al.* (11). Thicker black bars represent gRNA anchoring regions while hashed gray bars represent coverage range of identified gRNA families. Underscores (_) are used to clarify the editing sites to which the numbers above the lines correspond (CYb ES580 and MURF2 ES440). AuG start codon are also underlined. Pre-edited and fully edited sequences were excluded from IPS calculations. IPSs shown are present in at least 4 out of *n* = 5 replicates. Black diamonds represent IPSs present in all five replicates. Outlined diamonds represent IPSs present in four replicates. Small red u's denote uridines added to the sequence through editing, while large U’s denote uridines encoded by the mitochondrial genome. Green asterisks (*) denote encoded uridines that have been deleted. (**D**) Degree of pausing above threshold for identified IPSs. For each IPS, the total counts sharing the corresponding editing stop site were averaged and the fold count above the outlier threshold was calculated. Error bars represent one standard deviation.

We previously showed that in steady state populations of the pan-edited RPS12 and ND7-5′ mRNAs, exceedingly few or zero transcripts, respectively, correspond to the consensus fully edited sequence. Rather, the majority of transcripts are partially edited (8). Even considering transcripts that are edited through the start codon, and thus contain functional open reading frames with heterogeneous 5′ untranslated regions, <6% of RPS12 and ND7-5′ mRNAs can be considered fully edited. Thus, we first asked whether minimally edited mRNAs CYb and MURF2 exhibit similar ratios of pre-edited, fully edited and partially edited sequences as in pan-edited mRNAs. To this end, we isolated RNA from five replicates of PF strain 29-13 cells, and subjected equal amounts of RNA from each sample to RT-PCR within the linear range using primers corresponding to 5′ and 3′ never edited sequence flanking the edited regions. Amplicons were then sequenced using paired-end Illumina sequencing followed by TREAT analysis, during which we removed any sequences with non-T mismatches and normalized the resulting read counts to 100 000 for each sample as previously described (8) (‘Materials and Methods’ section). We then quantified the average normalized counts of pre-edited, partially edited and fully edited sequences for both CYb and MURF2, and identified similar patterns for both mRNAs (Figure 1A and Supplementary Table S2). Partially edited mRNAs are the most abundant category of mRNA, and pre-edited mRNAs comprise 22–27% of sequences. However, transcripts containing the published fully edited mRNA sequences (30) comprise 23.2% of CYb sequences and 29.7% of MURF2 sequences, levels that are significantly higher than those observed for either RPS12 or ND7-5′ mRNAs. One possibility is that the degree of full editing of the different mRNAs is differentially regulated. Alternatively, because the regions of CYb and MURF2 mRNAs requiring editing is much smaller than those in pan-edited mRNAs, requiring only two gRNAs each, the higher percentage of fully edited transcripts may simply reflect the effort needed to complete editing through two gRNAs compared to the five to nine gRNAs needed to generate fully edited ND7-5′ and RPS12. To address this possibility, we quantified the numbers of sequences edited correctly up to the end of the second gRNA from our previously published RPS12 and ND7-5′ datasets (8). We averaged the number of sequences canonically edited through ES40 in RPS12 and ES44 in ND7-5′ and compared those numbers to the proportion of fully edited sequence in CYb and MURF2. Sequences correctly edited up to the end of the second gRNA comprise 37.7% of total RPS12 sequences and 23.9% of ND7-5′ sequences, levels similar to the percentage of fully edited sequences for CYb and MURF2 (Figure 1B). Therefore, overall editing of the two minimally edited mRNAs is no more efficient than the editing of pan-edited mRNAs up to the second gRNA. Instead, the smaller region requiring editing allows a higher percentage of minimally edited transcripts to reach full editing.

Next, we asked whether there are intrinsic barriers to editing of minimally edited mRNAs as observed in the editing of pan-edited mRNAs. To analyze the extent of editing in partially edited transcripts, we define Editing Sites (ESs; Table 1) as any space between two non-U nucleotides and as such a potential site for U insertions or deletions. Editing proceeds in a 3′ to 5′ direction along an mRNA, and an Editing Stop Site is the 5′ most ES for which the sequence of a given transcript matches the canonical fully edited sequence (Table 1). As previously described (8), IPSs are defined as Editing Stop Sites whose magnitude is great enough to render them outliers relative to non-pause sites (Table 1). Statistically, outliers in a normal distribution are defined as those points >1.5 times the interquartile range (IQR) above the third quartile (3Q) (29). Thus, outliers in our data are defined as those Editing Stop Sites at which the total number of sequences exceeds the outlier threshold of (1.5 * IQR) + 3Q.

In general, IPSs may be bounded on their 5′ ends by either pre-edited or non-canonically edited junction sequence. Junctions are variable edited sequences that match neither pre-edited nor fully edited sequence and are present in partially edited mRNAs between the 3′ fully edited and 5′ pre-edited regions (31). The abundant presence of junction sequences in numerous kinetoplastid species has been known for more than two decades and was recently confirmed by high-throughput methods (8,32–36). The exceptions to IPSs being bounded at their 5′ ends by pre-edited sequence are those IPSs at the first editing site of an mRNA where, by definition, a pause followed by pre-edited sequence represents an unedited transcript. Unedited transcripts are excluded from the IPS calculation. Thus, IPSs at the first ES are always 5′ bounded by junction sequence.

In both RPS12 and ND7-5′ mRNAs, we previously identified IPSs interspersed throughout the lengths of gRNAs, representing pauses in canonical editing as a gRNA is being utilized and suggesting bottlenecks in mRNA/gRNA alignment and/or folding (8). To ask whether a similar pattern is evident in minimally edited mRNAs, we determined the IPSs for CYb and MURF2 mRNAs. We acknowledge that the gRNA populations for the specific cell lines used here have not been sequenced, nor have the specific interactions between gRNAs and cognate mRNA been determined experimentally. However, we recently provided evidence that the positions of gRNA populations are conserved between strains (9). Moreover, a comparison of gRNA populations from three *T. brucei* strains (11,37) reveals that CYb gRNA families are highly similar and primarily anchor at the same location, and the gMURF2 sequence is almost identical in all three *T. brucei* strains examined. Thus, we feel confident in interpreting CYb and MURF2 IPSs in the context of the published cognate gRNA sequences.

The positions of those IPSs identified in at least four out of the five replicates for each mRNA are shown in Figure 1C, aligned with the positions of cognate gRNAs. Quantification of IPSs is presented in Figure 1D. For CYb, two IPSs were present in all 5 replicates (Figure 1C, top). In contrast to the IPSs found in pan-edited mRNAs RPS12 and ND7-5′, CYb IPSs are not located in the middle of the gRNAs but instead are found at the end of the anchor region of the first gRNA (ES558) and at the exchange point between gRNA-1 and gRNA-2 (ES569). For MURF2, we found IPSs in all five replicates at the sites at which the first two Us are inserted (ES441 and ES446), which may represent the first and last editing sites specified by an unidentified gRNA (Figure 1C, bottom). We also identified a MURF2 IPS at the ES immediately 5′ of the start codon (ES457) in all but one replicate. These data lead us to conclude that minimally edited mRNAs have fewer barriers to gRNA utilization than do pan-edited mRNAs. Instead, the barriers to editing of minimally edited mRNAs appear to be primarily at the levels of commencing proper utilization of a gRNA and at the gRNA exchange step.

### CYb IPSs suggest multiple mechanisms of junction formation

**Figure 2.**
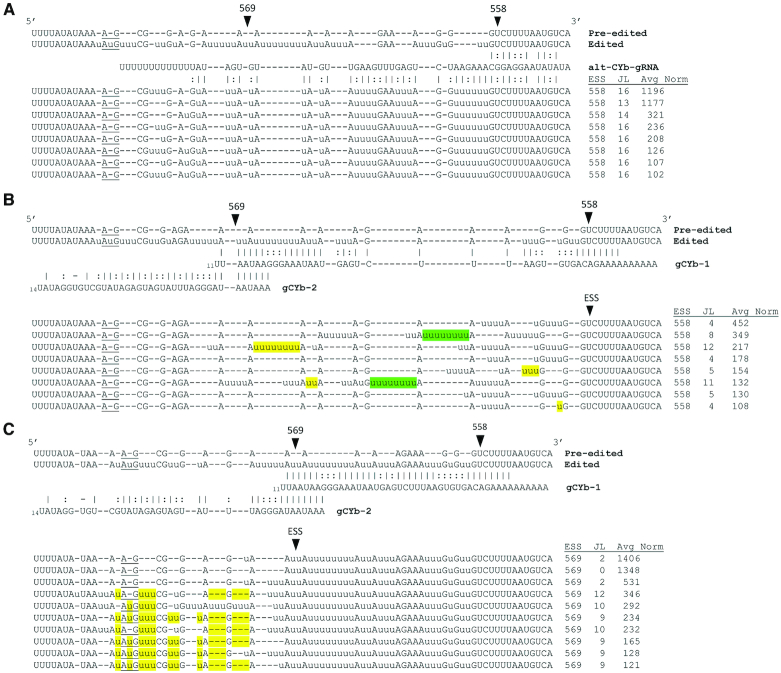
Sequence analysis of the most abundant junctions arising at IPSs in CYb mRNAs. (**A**) A family of junction sequences at Editing Stop Site (ESS)558 whose sequences suggest editing by an alternative gRNA. Above this sequence family, CYb pre-edited and fully edited mRNA sequences are aligned with an alternative gRNA, alt-gRNA-CYb, that could direct this editing pattern (top). Average normalized counts (Avg Norm) and lengths of junction sequences (JL) are indicated. Dashes (-) are used to align the non-U nucleotides for readability. AuG start codons (or their corresponding pre-edited positions) are underlined. (**B**) Most abundant junctions arising at ESS558 (>100 average normalized count) aside from those shown in (A). gRNA sequences as identified by Koslowsky *et al.* (11) are shown. Editing sites that match the canonical number of U’s are highlighted in yellow. Editing sites other than ES568 with 8 U’s are highlighted in green. (**C**) Most abundant junctions arising at ESS569 (>100 average normalized count). Editing sites that match the canonical number of U’s are highlighted in yellow.

mRNAs sharing the same IPS typically have highly heterogenous sequences 5′ of the IPSs, and the characteristics of these sequences can inform the nature of the pause in canonical editing. To understand pauses in CYb mRNA editing, we examined the top sequences arising at both IPSs, which occur at ES558 and ES569. To this end, we calculated the average normalized count of each unique sequence across the five replicates. Those sequences with an average normalized count of at least 100 are shown in Figure 2 and are grouped according to commonalities in their sequences. Sequences shown in Figure 2A and B comprise 37% of sequences that have entered the editing pathway and have Editing Stop Site 558, and those in Figure 2C represent 41.5% of total sequences with Editing Stop Sites 569. ES558 is the 5′ most site within the CYb anchor sequence, and is a site that does not require editing. Sequences with no junction at this site are pre-edited by definition and are not shown in Figure 2. An IPS at ES558 represents a buildup of sequences with junctions, and thus reveals a failure of the editing machinery to insert the canonical number of Us in this region, despite the occurrence of some editing activity (Figure 2A and B). One of the most abundant sequence families, comprising an average of about 5% of all total CYb sequences and ∼35% of partially edited sequences with Editing Stop Site 558, share a common non-canonical sequence for 13 ESs 5′ of this IPS (Figure 2A). The consensus region shared by these mRNAs is roughly the expected size directed by one gRNA, and these sequences have variable sequence at their 5′ ends that could represent a ‘true’ junction. Investigation of the gRNA database constructed by Koslowsky *et al.* (11) revealed a previously unidentified family of gRNAs that could direct this edited sequence. Shown in Figure 2A is the most abundant member of this gRNA family, which is also the one that harbors the least mismatches, although the predicted anchor region does contain G:U basepairing and one mismatch (alt-CYb-gRNA). Given the abundance of the alternative CYb mRNA sequence shown in Figure 2A, our data suggest that alt-CYb-gRNA is utilized with some frequency, even in the absence of complete Watson–Crick basepairing within the anchor duplex. Translation of this alternative edited mRNA sequence in the context of the rest of the CYb gene in all three reading frames failed to identify an open reading frame that incorporates edited sequence. Thus, this sequence family may represent a dead end population. Alternatively, these sequences could be editing intermediates, which eventually become ‘rewritten’ by the canonical initiating gRNA.

The remaining sequences with Editing Stop Site 558 and totaling greater than 100 normalized counts are shown in Figure 2B. These mRNAs contain mis-editing at between 4 and 12 editing sites 5′ of the anchor region of the initiating gRNA. While they have some commonalities, such as the lack of U addition at ES559, these sequences are generally heterogeneous. We did note that ES568 contains 8 Us in the canonical sequence (Figure 2B, yellow), and two abundant junction sequences have 8 Us added at other sites (ES563 and ES564; Figure 2B, green). Given that we do not typically observe abundant large stretches of U insertions in junction regions and that gCYb-1 directs the addition of 8 Us at a specific site, these sequences likely arose through mis-alignment of the canonical initiating gRNA.

Finally, ES569 is the site of gRNA exchange, based on the published list of gRNAs directing CYb mRNA editing (11,37) (Figure 2C). Partially edited CYb mRNA sequences with Editing Stop Site 569 are edited correctly up to the last site directed by the initiating gRNA. The highest abundance sequences at this Editing Stop Site have very short or no junctions, perhaps due to the absence of a stably associated gCYb-2. The remaining top junctions are longer (9–12 ESs) and appear to be intermediates which struggle to insert the full 5 Us required at ES570, having 2 or 3 Us inserted instead. Together, analysis of junctions at IPSs in CYb mRNAs suggests that pauses in canonical edited sequences arise through multiple mechanisms including incorrect gRNA utilization, misalignment of the canonical gRNA, and inefficient gRNA anchoring.

### Characterization of MURF2 IPSs

**Figure 3.**
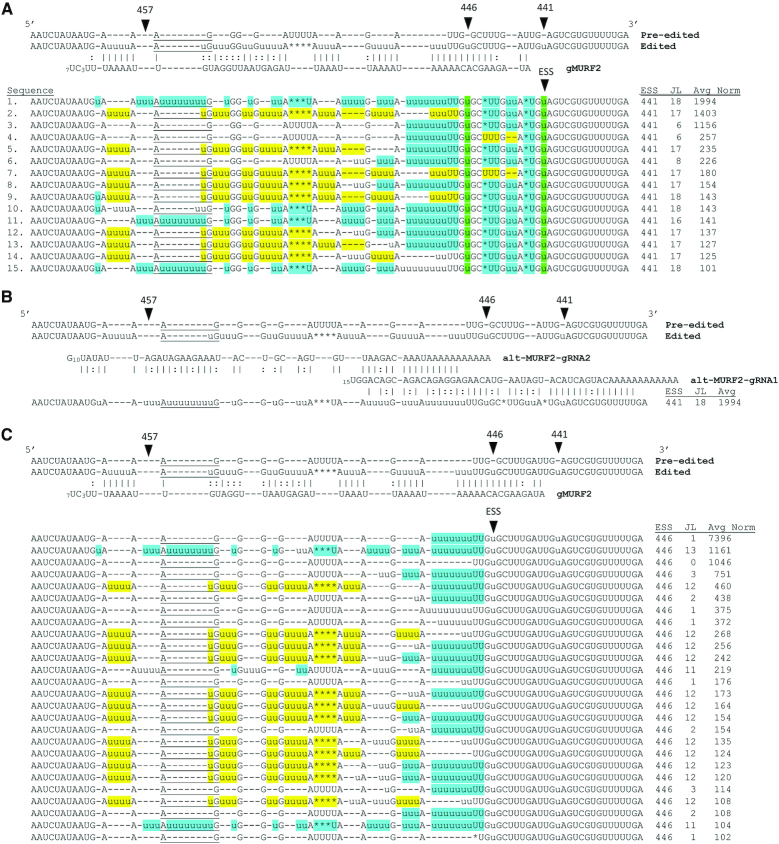
Sequence analysis of the most abundant junctions arising at IPSs in MURF2. (**A**) Most abundant junctions arising at ESS441 (>100 average normalized count). MURF2 pre-edited and fully edited sequence, along with the gRNA sequence identified by Koslowsky *et al.* (11) as directing this sequence are shown above. Average normalized counts (Avg Norm) and lengths of junction sequences (JL) are shown below. Editing sites that match the canonical number of u's are highlighted in yellow, while editing sites matching modification by alternative gRNAs, alt-MURF2-gRNA1 and alt-MURF2-gRNA2, are highlighted in blue. Editing sites that are canonically edited and whose editing can also be directed by the alternative gRNA (alt-MURF2-gRNA1) shown in (**B**) are highlighted in green. Dashes (-) are used to align the non-U nucleotides for readability. AuG start codons (or their corresponding pre-edited positions) are underlined. (B) Alternative gRNAs that could direct the editing of the most abundant junction at ESS441. (**C**) Most abundant junctions arising at ESS446 (>100 average normalized count).

Next, we examined the most abundant junctions arising at IPSs in MURF2 mRNA to identify factors that contribute to pauses in editing of this mRNA. Analysis of MURF2 sequences is a challenge because only one gRNA directing this region has been identified (hereafter referred to gMURF2) (11). No gRNA has yet been identified that could direct the addition of Us at ES441 and ES446. Figure 3A shows the most abundant junctions (>100 average normalized count) arising following Editing Stop Site 441. These sequences represent 48% of the total sequences with this Editing Stop Site. Strikingly, a majority of these sequences are similar in that they contain single U deletions at ES442 and ES444, and the addition of two Us at ES443, generating a sequence quite different than canonical edited MURF2. Moreover, 51% of mRNAs with this editing pattern also contain 9 Us at ES447 rather than the canonical 5 Us. Because the majority of top junctions arising at ES441 contain this extended pattern of modifications, we reasoned that the observed differences from canonical fully edited MURF2 sequence could have arisen by utilization of an alternate or incorrect gRNA. Again searching the gRNA database constructed by Koslowsky, *et al.* (11), we identified two gRNAs that could direct these modifications (Figure 3B). The first gRNA (alt-MURF2-gRNA1), which has not previously been identified, can anneal to the mRNA downstream of ES441 and direct the U deletions at ES442 and ES444 as well as the two U additions at ES443 and the 9 U additions at ES447. This gRNA also directs the canonical single U additions at ES441 and ES446. A second, less abundant gRNA (alt-MURF2-gRNA2) can anneal at ES447 and direct the editing of a longer non-canonical junction sequence observed in the most abundant junction following Editing Stop Site 441 (Figure 3A, sequence 1; Figure 3B). Utilization of alt-MURF2-gRNA2 appears to be promiscuous, as this gRNA was previously identified as directing editing of CR4 mRNA in the region of nt 244–295 (11).

While the extended junction sequence directed by sequential usage of alt-MURF2-gRNA1 and alt-MURF2-gRNA2 is the most abundant single junction at Editing Stop Site 441, when all junctions at this site are considered, it is less common than the combined junctions containing alternative sequence at ES442-444 and then returning to the canonical sequence at a more 5′ site (Figure 3A). Some of the latter sequences are consistent with gMURF2 anchoring to alternative sequence and then bypassing ES447 or ES447/448 before directing canonical editing at more 5′ sites (see sequences 5, 8, 12 and 13 in Figure 3A). Importantly, other sequences are consistent with canonical gMURF2-directed re-editing of some of alternatively edited sites. For example, sequences 2, 7 and 9 (Figure 3A) contain alternative sequence at ES442-444, but ES447 and ES448 appear to have been remodeled by the canonical gRNA and followed by additional 5′ canonical editing.

For those MURF2 mRNAs with Editing Stop Site 446 (Figure 3C), the most abundant junction consists of 9 Us at ES447 and no other modifications 5′ of this site (JL 1, top line, Figure 3C). Indeed, these mRNAs comprise over 7% of the total MURF2 mRNA population. The 9 Us at ES447 in this class of mRNAs were likely not directed by alt-MURF2-gRNA1, as these mRNAs are canonically edited 3′ of ES447 (by definition of Editing Stop Site 446). A plausible explanation for their generation is misalignment of gMURF2 to direct insertion of 9 Us at ES447 following canonical editing up to this point. A bulge of one U in the gMURF2 would leave a run of 9 As to direct the observed U insertions at ES447. The observation that we identified no mRNAs with both the canonical 4 Us at ES448 and 9 Us at ES447 in our population (Figure 3A) supports the misalignment model for this population of mRNAs. Those mRNAs with Editing Stop Site 446 and 9 Us at ES447 can apparently be further edited by either gMURF2 or alt-MURF2-gRNA2 or (see sequences with JL 11-13; Figure 3C). The complex pattern of editing that results in a ‘hybrid’ mRNA with non-canonical sequence at ES447 or ES447/448 flanked both 3′ and 5′ by canonically edited sequence could be explained by misalignment of gMURF2 following canonical editing by the unidentified downstream gRNA as described above. However, both the single 9 U insertion at ES447 and the more complicated ‘hybrid’ mRNAs are also consistent with re-editing of alternatively edited sequence at ES442-444 or utilization of an unidentified gRNA encoding ‘hybrid’ canonical/alternative sequence in this region. Our data cannot resolve these possibilities. Overall, we show that editing of MURF2 mRNA is substantially more complex than previously appreciated and may involve more than one unidentified gRNA. We also note that a subpopulation of the mRNAs shown in Figure 3C with junctions between ES447-ES450 and long stretches of 5′ canonical editing can be *in silico* translated into open reading frames with minimal conserved amino acid changes compared to the canonical MURF2 sequence.

The final IPS identified in MURF2 mRNA is ES457, one ES 5′ of the start codon (Figure 3). The most abundant junctions arising at ES457 contain (in order of highest to lowest normalized sequence count) three, two, none and one uridine at ES458 instead of the canonical four. This variation in the 5′ UTR sequence is similar to what was previously observed in RPS12 and ND7-5′ mRNAs (8), and suggests that 5′ UTR variation will continue to be identified as a hallmark of edited mRNAs.

### Effects of ablation of editing accessory factors on CYb and MURF2 edited mRNA populations

Having identified the intrinsic features of CYb and MURF2 mRNA editing, we set out to evaluate the roles of known editing accessory factors on the editing of these mRNAs. Early studies of PF *T. brucei* using poisoned primer extension showed that MRP1/2 and RBP16 primarily affect CYb and MURF2 mRNA editing, with additional effects on stability of some never-edited RNAs (17,20). Fisk *et al.* (19), using qRT-PCR, showed that depletion of either MRP1/2 or RBP16 results in a greater than 80% decrease in fully edited CYb with little or no apparent effect on pre-edited CYb mRNA levels. With regard to MURF2, MRP1/2 depletion led to a dramatic reduction in edited MURF2 mRNA and a modest reduction in pre-edited MURF2. RBP16 knockdown caused a modest decrease in edited MURF2 mRNA and left pre-edited mRNA unaffected. Due to the necessity of designing qRT-PCR primers that impinge on the editing domains of these mRNAs, previous results could be skewed due to the limitations of primer design. For example, a qRT-PCR primer targeting pre-edited mRNA could anneal to an mRNA that contains a small sequence change, but is still similar enough to sufficiently anneal to the primer. TREAT analysis allows us to examine the entire editing domains of these two minimally edited mRNAs in the absence of any sequence bias. TREAT also permits us to evaluate the step(s) at which different factors impact the editing process at much higher resolution than previous methods (9,10).

**Figure 4.**
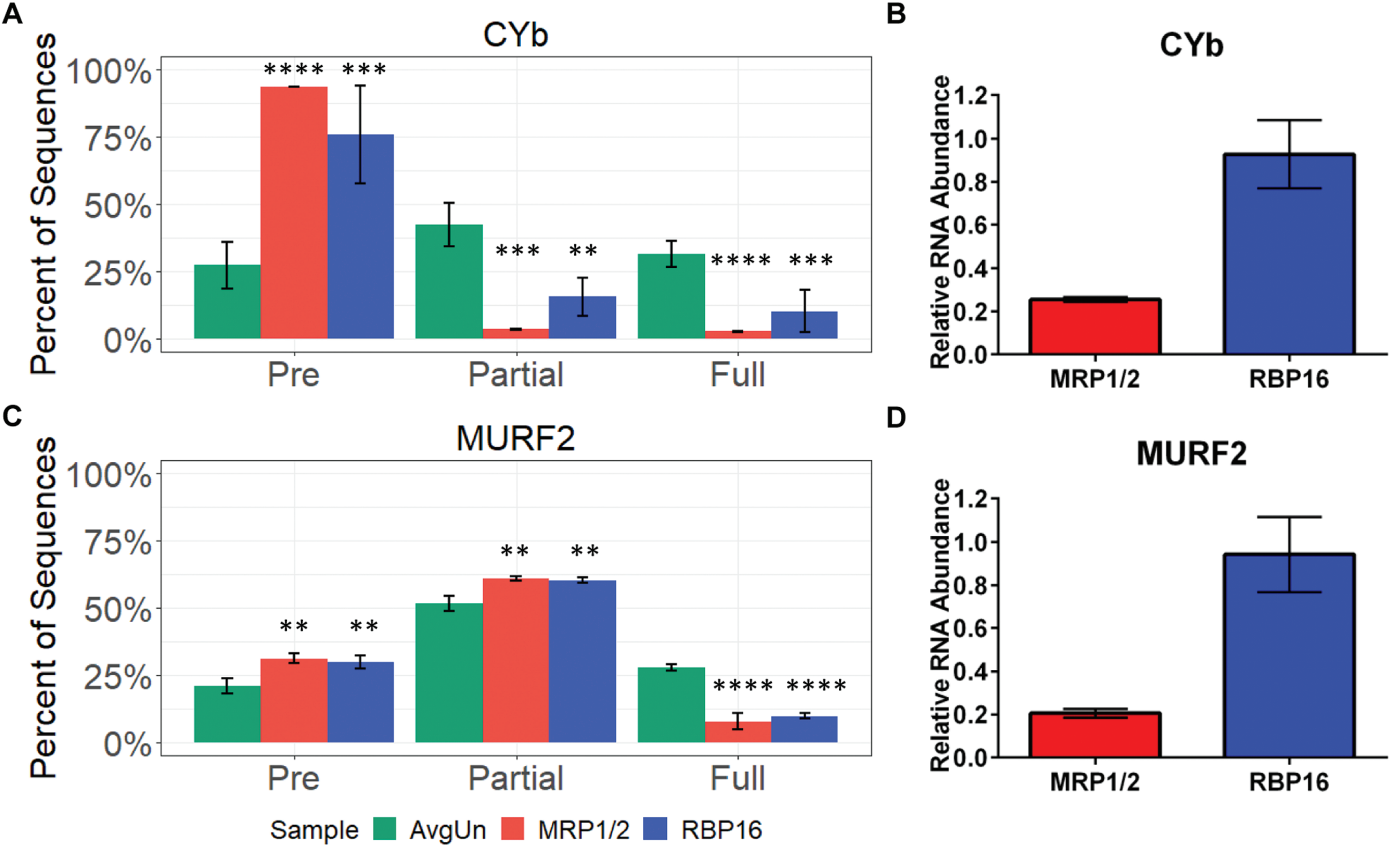
Effect of RBP16 and MRP1/2 depletion on editing and stability of CYb and MURF2 mRNAs. (**A**) The percentage of normalized pre-edited, partially edited and fully edited CYb sequences in uninduced controls (AvgUn) and MRP1/2 or RBP16 knockdown cells. Uninduced cells represent an average of all uninduced lines in this study (*n* = 8); RNAi induced MRP1/2 or RBP16 cells represent *n* = 2 for each cell line. Error bars represent one standard deviation. *P < 0.05; **P < 0.01; ***P < 0.001; ****P < 0.0001 (Student's *t*-test). (**B**) Total CYb mRNA levels in RNAi induced cells relative to uninduced cells were determined by qRT-PCR (*n* = 6). (**C** and **D**) As above in (A and B) for MURF2 mRNA.

To investigate the roles of RBP16 and MRP1/2 in CYb and MURF2 mRNA editing with TREAT, we used previously constructed cell lines containing a tetracycline-inducible RNAi system to knock down expression of either RBP16 or MRP1/2 (17,19). We then performed high-throughput sequencing on two biological replicates each of the RNAi cell lines after 3 days of growth in either uninduced or tetracycline-induced conditions. At this time point, a modest growth defect was evident, with RPB16 and MRP1/2 cells at an average of 44 and 74% of wild-type levels, respectively (Supplementary Figure S1). In these cells, qRT-PCR showed that MRP1 mRNA was reduced an average of 70% and RBP16 mRNA was reduced an average of 95% compared to uninduced controls, and western blot analysis revealed a very substantial decrease in RBP16 and MRP2 proteins (Supplementary Figure S1). To begin, we utilized high-throughput sequencing and TREAT analysis to compare the levels of pre-edited, partially edited and fully edited transcripts in cells depleted of RBP16 or MRP1/2 to the average of those in the eight uninduced controls used in this study. MRP1/2 knockdown resulted in an almost complete absence of fully or partially edited CYb mRNAs; pre-edited mRNA constituted 94% of the total CYb population in MRP1/2 depleted cells compared to 27% in uninduced cell lines (Figure 4A, red). A similar, although slightly lessened effect was observed when RBP16 was knocked down (Figure 4A, blue). Thus, ablation of either MRP1/2 or RBP16 leads to a significant decrease in the proportion of both partially and fully edited CYb mRNA and a corresponding increase in the proportion of fully edited mRNA.

Because our high-throughput sequence analysis includes a normalization of total read counts (after deleting those with non-T mismatches; Supplementary Table S2) in all samples to 100 000, these results could have arisen in two different ways. First, the large increases in pre-edited CYb mRNA observed upon MRP1/2 and RBP16 knockdown could reflect unchanged total CYb mRNA levels and failure of the entire mRNA population to enter the editing pathway. Alternatively, if the level of total CYb mRNA is decreased, the data in Figure 4A could reflect relatively static pre-edited mRNA levels and degradation of partially and fully edited CYb mRNAs. To distinguish between these possibilities, we quantified the levels of total CYb mRNA by qRT-PCR using primers against the never-edited region of CYb. While total CYb levels were essentially unchanged upon RBP16 knockdown (Figure 4B, blue), we observed a 75% reduction of CYb transcripts in the MRP1/2 knockdown (Figure 4B, red). This finding indicates that entry of CYb mRNA into the editing pathway is strongly impeded in the absence of RBP16. For MRP1/2 depleted cells, it may also be the case that entry of CYb mRNA into the editing pathway is blocked and that excess pre-edited mRNA is degraded. On the other hand, it is striking that approximately 25% of total CYb mRNA is pre-edited in uninduced cells (Figure 4A, green), and that ∼25% of total CYb mRNA remains following MRP1/2 depletion (Figure 4B, red). These data are more consistent with a model in which those CYb mRNAs that have entered the editing pathway are rapidly degraded in MRP1/2 depleted cells, implicating MRP1/2 as a stabilizing factor for edited CYb mRNA. Collectively, these data demonstrate that accessory editing factors RBP16 and MRP1/2 promote increased levels of edited CYb mRNA in PF *T. brucei* using different mechanisms.

We next examined the effects of ablating MRP1/2 and RBP16 on MURF2 mRNA populations using high-throughput sequencing, and found that fully edited MURF2 mRNA decreased from approximately 25% of total to 8–10% of total sequences in both knockdowns (Figure 4C). However, unlike CYb mRNA, both pre-edited and partially edited MURF2 mRNAs were significantly increased upon knockdown of either MRP1/2 or RBP16 (Figure 4C). qRT-PCR targeting the never edited region of MURF2 revealed no change in total MURF2 mRNA upon RBP16 knockdown and an 80% reduction in total MURF2 mRNA levels upon MRP1/2 knockdown (Figure 4D). Thus, MRP1/2 acts as a stabilizing factor for both minimally edited mRNAs, although its effects on MURF2 mRNA differ from its effects on CYb mRNA in that partially edited MURF2 mRNAs do not require MRP1/2 for their stabilization. Beyond the stabilizing effect of MRP1/2, the increased pre-edited and partially edited MURF2 mRNA populations support functions for MRP1/2 and RBP16 in MURF2 mRNA editing at both initiation and progression steps (Figure 4C; and see below). Therefore, the roles of MRP1/2 and RBP16 in editing differ between the two minimally edited mRNAs examined here.

To define in detail the effects of MRP1/2 and RBP16 on editing progression in minimally edited mRNAs, we used TREAT to analyze the partially edited mRNA populations. We calculated EPSs as described previously (9) (Table 1). Briefly, we quantify the Editing Stop Sites that increase significantly (*P* < 0.05, q < 0.05) upon depletion of a given factor relative to uninduced controls, and we define EPSs as sites at which Editing Stop Sites are significantly increased in both replicates. Upon initial analysis of the CYb mRNA population we observed no EPSs for either knockdown line, indicating that neither MRP1/2 nor RBP16 significantly affect the 3′ to 5′ progression of editing of the overall mRNA population. However, given the high numbers of pre-edited CYb sequences in our knockdown samples (Figure 4), we wondered whether small but specific effects on progression were masked by low numbers of partially edited CYb sequences relative to those in the uninduced samples. In order to effectively compare only transcripts that have entered the editing pathway, and thus examine the mechanistic effects of MRP1/2 and RBP16 knockdown on editing progression, we removed pre-edited sequences from both our uninduced and induced sample sets and renormalized the remaining sequences to 100 000. Performing our analysis on this new dataset, we again identified no EPSs upon either MRP1/2 or RBP16 depletion. Thus, we conclude that neither MRP1/2 nor RBP16 play a significant role in CYb editing progression.

**Figure 5.**
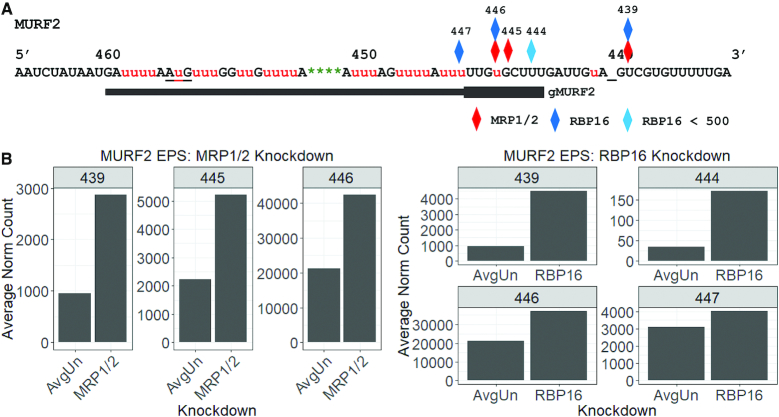
EPSs in MURF2 mRNA resulting from RBP16 and MRP1/2 depletion. (**A**) MURF2 edited mRNA sequence with EPSs determined by comparison of induced knockdown cells (*n* = 2 for each cell line) to controls comprised of all uninduced cell lines used in this study (*n* = 8) after removal of pre-edited sequences and renormalization. Diamonds represent the locations of EPSs present in both replicates in each knockdown cell line. Lighter colored diamonds represent EPSs at which both uninduced and induced replicates have <500 average renormalized count. The position of gMURF2 is shown; thick region indicates anchor region. Underscore (_) clarifies the position of ES440. The AuG start codon is also underlined. (**B**) Average number of sequences at each EPS for MRP1/2 knockdowns (left) and RBP16 knockdowns (right), and their uninduced controls (AvgUn).

We next turned to the effects of MRP1/2 and RBP16 on MURF2 mRNA editing progression, considering the canonical MURF2 edited sequence and again using a renormalized dataset containing only those mRNAs that had entered the editing pathway. We identified four EPSs that arise upon RBP16 knockdown and three EPSs that arise upon MRP1/2 knockdown, two of which occur at the same sites (Figure 5). The majority of the EPSs in both knockdowns occur within the region whose editing is presumed to be directed by an unidentified gRNA (ES439-446). In addition, one EPS identified in both knockdowns occurs at a site within the predicted anchor region of the unidentified gRNA (ES439). mRNAs with an EPS at ES439 are, by definition, edited at ES440, which is downstream of the first canonical editing site. Our initial analysis of junction sequences at this site in both MRP1/2 and RBP16 knockdowns reveals long junctions, many of which appear to have identical regions of editing (Supplementary Figure S2). This may indicate increased usage of an unidentified alternative gRNA upon knockdown of either MRP1/2 or RBP16. Overall, editing of MURF2 mRNA at ES439, while relatively infrequent, suggests an effect of both proteins on anchor duplex formation between the mRNA and the initiating gRNA, with the loss of the proteins resulting in increased ‘breathing’ of mRNA/gRNA pairs that permits U insertion within the canonical anchor region of the mRNA.

We also identified a highly significant and abundant pause in MURF2 editing progression at ES446 in both MRP1/2 and RBP16 knockdowns (Figure 5). These mRNAs represent those with the first two Us (at ES441 and ES446) correctly inserted, but for which editing directed by gMURF2 has not correctly initiated. Analysis of the most abundant junctions at this EPS in MRP1/2 and RBP16 knockdowns reveals a heterogeneous majority of junctions in both knockdowns with 9 Us at ES447 and either short (JL1-3) or long (JL 11-13) junctions. Examining those mRNAs with long junctions (Supplementary Figure S3), we observe increased numbers of ‘hybrid’ junctions (those with 9 Us at ES447 and canonical editing both upstream and downstream of that site as discussed above for the MURF2 mRNA population in wild-type cells) in both knockdowns, with the greatest increase observed upon knockdown of RBP16 (Supplementary Figure S3B). Overall, we observed significant pausing within MURF2 mRNA upon both MRP1/2 and RBP16 knockdown, with sites identified in both the anchor duplex and gRNA directed regions. These data suggest that both factors promote correct gRNA-mRNA alignment, notably at ES447 and 448 where gRNA misalignment may lead to an increase in ‘hybrid’ mRNAs. Additionally, we note that the pattern of exacerbated pausing is similar in the MRP1/2 and RBP16 RNAi lines in both the levels of pausing and in junction sequences themselves, suggesting that these two editing accessory factors perform related roles in the progression of editing through MURF2 mRNA.

### Effects of ablation of RESC factors on CYb and MURF2 edited mRNA populations

Having investigated the roles of accessory factors on the editing of minimally edited mRNAs, we next looked at the roles of two components RESC: TbRGG2, a central component of the REMC and GAP1/2, a heterotetramer that comprises a component of the Guide RNA Binding Complex. GAP1/2 also forms additional complexes with other proteins involved in mRNA editing and stability (10,38,39). Both TbRGG2 and GAP1/2 are essential for editing (13–16). TbRGG2 impacts the 3′ to 5′ progression of editing along pan-edited mRNAs (9,12), whereas qRT-PCR studies suggest little impact of this protein on minimally edited mRNAs (13,14). The GAP1/2 heterotetrameric complex, on the other hand, is essential for gRNA stability, and depletion of GAP1/2 causes a decrease in fully edited transcripts and a concomitant 2- to 5-fold increase of most pre-edited transcripts (15,16). To examine the roles of TbRGG2 and GAP1/2 in editing in more detail, we performed high-throughput sequencing and subsequent TREAT analysis on CYb and MURF2 cDNA derived from two biological replicates of each RNAi cell line following 3 days of tetracycline induction, a time at which growth defects had not yet manifested (Supplementary Figure S1) (13,16). TbRGG2 and GAP1 mRNAs were decreased an average 80 and 75%, respectively, compared to levels in uninduced cells, and western blot analysis demonstrated that both TbRGG2 and GAP1 protein levels were substantially decreased (Supplementary Figure S1).

**Figure 6.**
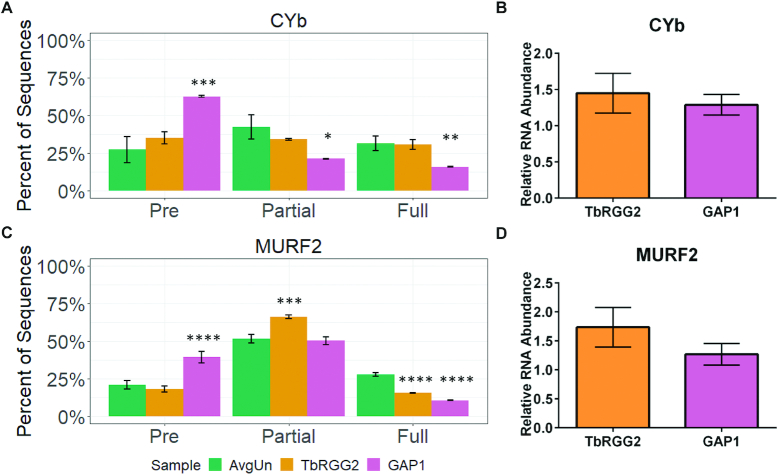
Effect of TbRGG2 and GAP1 depletion on editing and stability of CYb and MURF2 mRNAs. (**A**) The percentage of normalized pre-edited, partially edited and fully edited CYb sequences in uninduced controls (AvgUn) and TbRGG2 or GAP1 knockdown cells. Uninduced cells represent an average of all uninduced lines in this study (*n* = 8); RNAi induced TbRGG2 or GAP1 cells represent *n* = 2 for each cell line. Error bars represent one standard deviation. *P < 0.05; **P < 0.01; ***P < 0.001; ****P < 0.0001 (Student's *t*-test). (**B**) Total CYb mRNA levels in RNAi induced cells relative to uninduced cells were determined by qRT-PCR (*n* = 6). (**C** and **D**) As in (A and B) for MURF2 mRNA.

To begin, we examined the effects of TbRGG2 and GAP1 knockdown on total CYb mRNA levels by qRT-PCR and showed total mRNA was slightly increased in both knockdowns (Figure 6B). Quantification using TREAT revealed an absence of any significant effect of TbRGG2 depletion on the proportions of pre-edited, partially edited, or fully edited mRNA levels, consistent with published qRT-PCR results (Figure 6A, orange). In contrast, GAP1 knockdown resulted in significantly decreased partially and fully edited CYb mRNAs and an increase in pre-edited CYb mRNA as expected for a gRNA stabilizing protein (Figure 6A, pink). We observed somewhat different effects of these factors when examining MURF2 mRNA (Figure 6C). Again, total MURF2 mRNAs were slightly increased in TbRGG2 and GAP1 depleted cells (Figure 6D). However, TbRGG2 knockdown led to a significant decrease in fully edited MURF2 mRNA relative to uninduced samples (Figure 6C, orange). Moreover, we observed no change in pre-edited mRNA levels, but rather saw a significant increase in partially edited mRNAs, implicating TbRGG2 in MURF2 editing progression (Figure 6C, orange). GAP1 knockdown resulted in significantly decreased fully edited and increased pre-edited levels of both CYb and MURF2 mRNAs (Figure 6A and C, pink). However, partially edited mRNA levels remained unchanged, revealing somewhat different effects of GAP1 on MURF2 editing compared to its effect on CYb (compare GAP1, Figure 6A and C).

**Figure 7.**
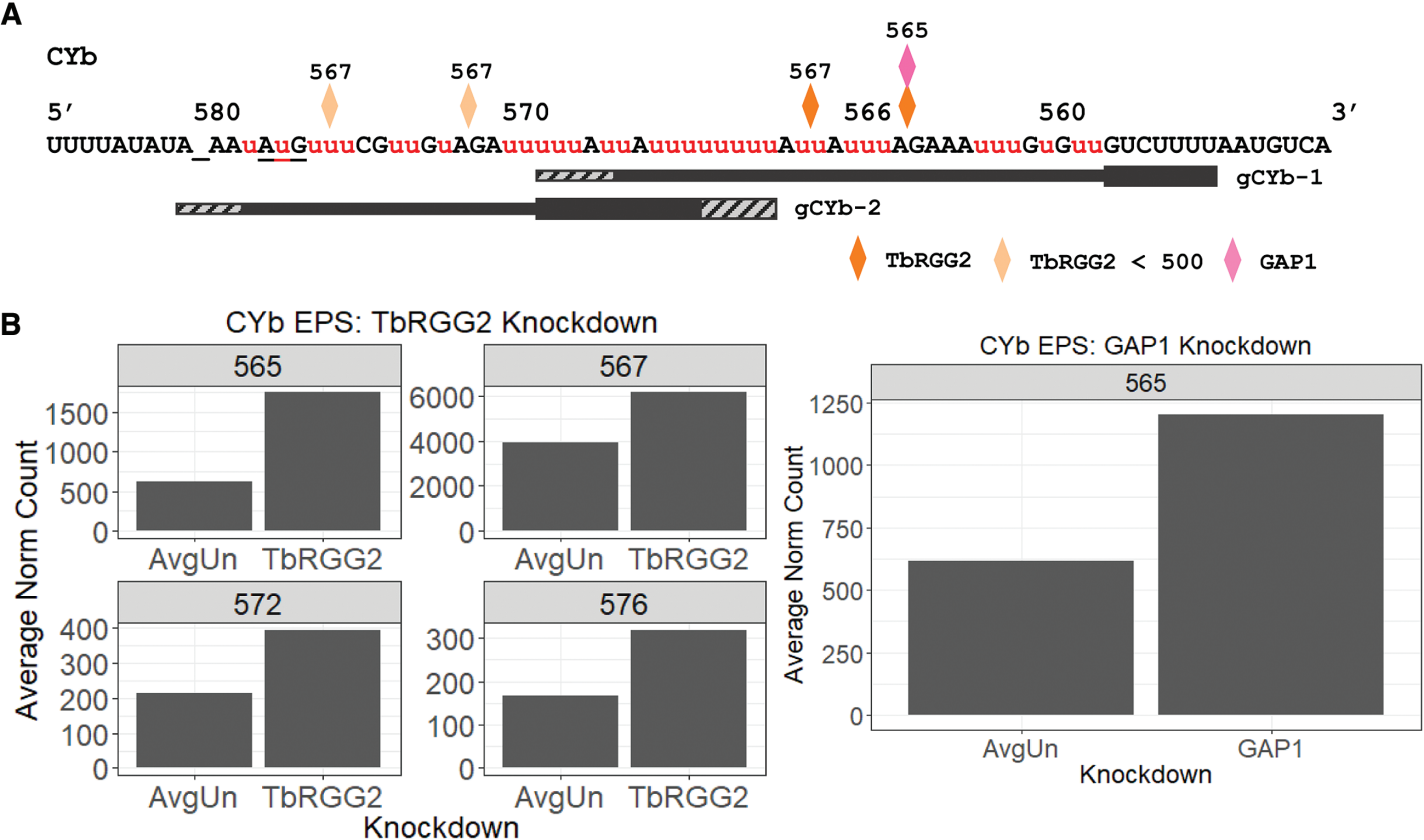
EPSs in CYb mRNA resulting from TbRGG2 and GAP1 depletion. (**A**) CYb edited mRNA sequence with EPSs determined by comparison of induced knockdown cells (*n* = 2 for each cell line) to controls comprised of all uninduced cell lines used in this study (*n* = 8) after removal of pre-edited sequences and renormalization. Diamonds represent the locations of EPSs present in both replicates in each knockdown cell line. Lighter colored diamonds represent EPSs at which both uninduced and induced replicates have <500 average renormalized count. The positions of CYb gRNAs are shown; thick regions indicate anchor regions and hatched regions indicate differences in gRNA families (11). Underscore (_) clarifies the position of ES580. The position of the AuG start codon is also underlined. (**B**) Average number of sequences at each EPS for TbRGG2 knockdowns (left) and GAP1 knockdowns (right), and their uninduced controls (AvgUn).

Next, to evaluate in greater detail the roles of TbRGG2 and GAP1 in the progression of editing of CYb and MURF2 mRNAs, we quantified EPSs after renormalizing the population of mRNAs that had entered the editing pathway as described above. Within CYb mRNA, we identified four EPSs arising from TbRGG2 depletion and one EPS due to GAP1 depletion (Figure 7A). Each of the TbRGG2 EPSs falls within gRNA-defined blocks, a feature which is a hallmark of a defect in gRNA utilization similar to what we observed with pan-edited mRNAs upon TbRGG2 knockdown (9). Two of the four EPSs, at ES565 and ES567, exhibit a total increase of over 1000 renormalized counts upon RNAi induction (Figure 7B) and we examined these sequences in more detail. For both of these EPSs, the pattern of junction sequences is similar to that in uninduced cells except that the most abundant sequence, which lacks a junction, increases from ∼65% in uninduced cells to ∼75% upon TbRGG2 depletion. Thus, although TbRGG2 knockdown does not have a major effect on junction lengths in the total CYb mRNA population (Supplementary Figure S4), we observe a modest increase in specific mRNAs lacking a junction, reminiscent of the overall increase in pan-edited mRNAs lacking junctions in TbRGG2 knockdowns (9). In the GAP1 knockdown, the EPS at ES565 also exhibits an increase in junction zero sequences from 65% in uninduced to 84% in induced cells. This finding of a GAP1 EPS within a gRNA block is unexpected, as GAP1 EPSs are almost exclusively found near the ends of gRNAs in RPS12 and ND7-5′ (9). These data may reflect the existence of GAP1 in defined complexes apart from fully assembled RESC, whose functions are not completely understood (10,38,39). Overall, these data lead us to conclude that TbRGG2 contributes modestly to the progression of CYb editing and that GAP1 may have additional roles in CYb editing beyond gRNA stabilization.

**Figure 8.**
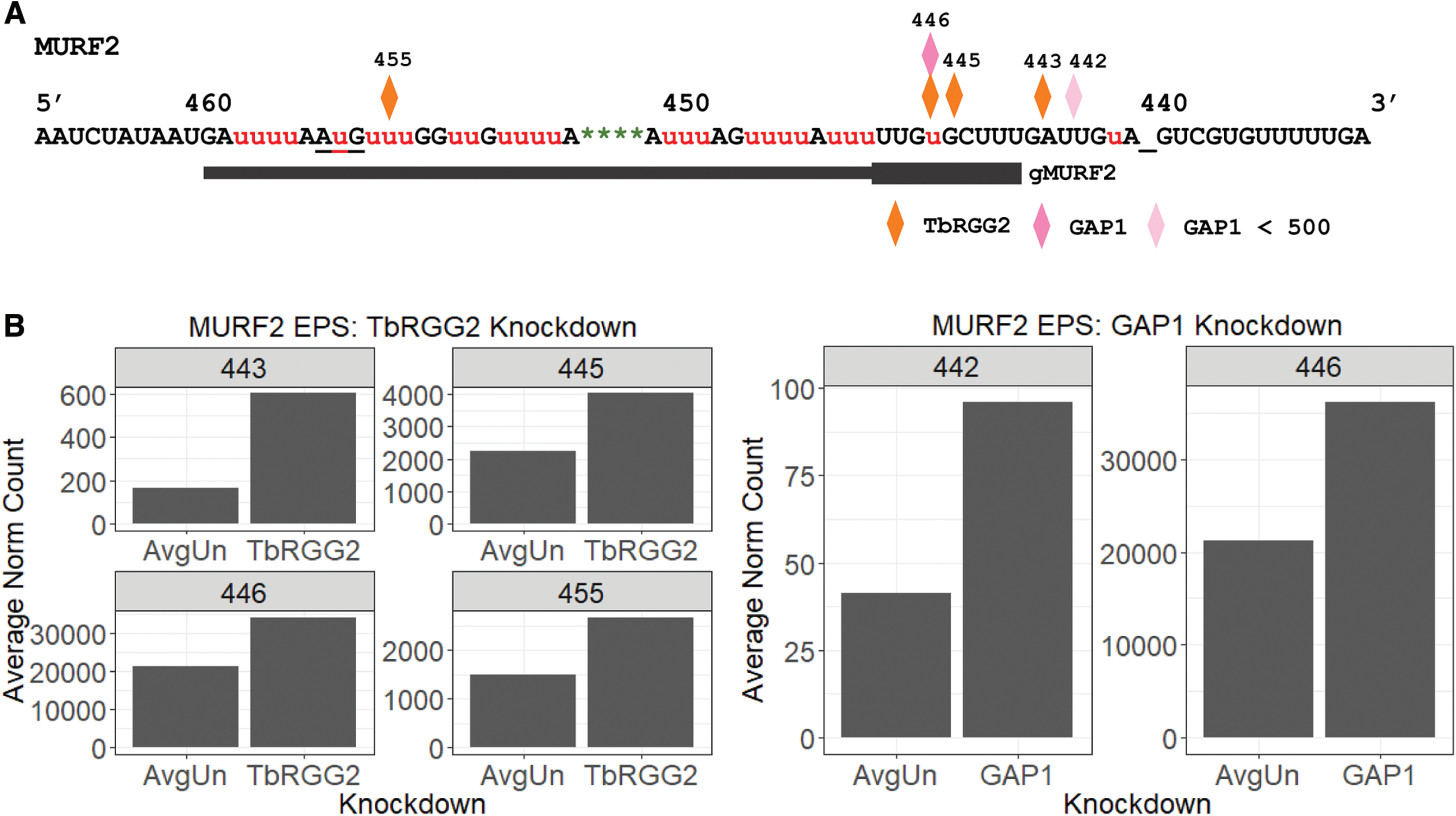
EPSs in MURF2 mRNA resulting from TbRGG2 and GAP1 depletion. (**A**) MURF2 edited mRNA sequence with EPSs determine by comparison of induced knockdown cells (*n* = 2 for each cell line) to controls comprised on all uninduced lines used in this study (*n* = 8) after of pre-edited sequences and renormalization. Diamonds represent the locations of EPSs present in both replicates in each knockdown cell line. Lighter colored diamonds represent EPSs at which both uninduced and induced replicates have <500 average renormalized counts. Underscore (_) clarifies the position of ES440. The AuG start codon is also underlined. (**B**) Average number of sequences at each EPS for TbRGG2 knockdowns (left) and GAP1 knockdowns (right), and their uninduced controls (AvgUn).

To define TbRGG2 and GAP1 effects on MURF2 mRNA editing in greater detail, we evaluated the EPSs arising from TbRGG2 and GAP1 knockdown in MURF2 mRNA. We found four EPSs for TbRGG2 and two EPSs for GAP1, and with the exception of TbRGG2 EPS 455, the EPSs all occur within the region directed by an as yet unidentified gRNA (Figure 8A). We also analyzed overall changes in junction lengths within each mRNA population and observed a significant increase in sequences lacking a junction or having short junctions (JL1-2) in the TbRGG2 knockdown, and a concomitant decrease in sequences with long junctions (JL11-13) (Supplementary Figure S4), similar to previously reported TbRGG2 effects on junction length (9). Of the EPSs that arise upon TbRGG2 and GAP1 knockdown, by far the most abundant is the EPS at ES446 (Figure 8B). Strikingly, ‘hybrid’ junctions with 9 Us in ES447 and canonical sequence upstream and downstream are absent in TbRGG2 depleted cells and dramatically reduced in prevalence in GAP1 depleted cells (Supplementary Figure S5A). If hybrid junctions arise from misalignment of gMURF2 (see above), these sequences may reflect a function of TbRGG2, and potentially GAP1, in modulating mRNA/gRNA interactions to promote non-linear editing that contributes to junction formation, consistent with reported TbRGG2 function in RPS12 mRNA editing (9). Finally, we quantified the abundance of sequences that are likely derived from usage of alt-MURF2-gRNA2 (Supplementary Figure S5B). Interestingly, while these represent the most abundant sequences in mRNAs with Editing Stop Site 446 and subsequent long junctions in uninduced cells, and in TbRGG2, MRP1/2 and RBP16 knockdowns, this is not the case in GAP1 knockdowns (Supplementary Figures S2 and 4A). The apparent decrease in alt-MURF2-gRNA2 usage in cells lacking GAP1 could either be a direct effect on gRNA selection, or considering that GAP1 is critical for gRNA stabilization, knockdown of GAP1 could lead to a disproportionate loss in alt-MURF2-gRNA2, which is much less abundant than gMURF2 ((11) and data not shown).

## DISCUSSION

Previous high-throughput sequencing studies of U insertion/deletion editing in *T. brucei* focused only on pan-edited mRNAs (8,10). Here, we use our TREAT bioinformatic platform to analyze two minimally edited mRNAs, thereby providing an opportunity to examine the mechanisms of editing within a region defined by 1–2 gRNAs. We examined CYb and MURF2 mRNA sequences in wild-type 29-13 cells as well as in RNAi induced knockdowns of several factors essential for their editing. We show that gRNA utilization in minimally edited mRNAs is more efficient than for pan-edited mRNAs, and we demonstrate multiple mechanisms of junction formation. Knockdown of editing factors revealed gene-specific functions for MRP1/2 and RBP16 in minimally edited mRNAs, modest effects of TbRGG2 on editing progression in CYb and MURF2 mRNAs, and a potentially novel function for GAP1 in editing progression.

One striking finding was that the proportions of fully edited CYb and MURF2 mRNAs in wild-type cells were much higher than those previously found for pan-edited mRNAs RPS12 and ND7-5′ (8). Upon re-examination of our published data, we found that the levels of pan-edited mRNAs that were edited to at least the end of the second gRNA were comparable to the levels of fully edited CYb and MURF2. Thus, abundant fully edited CYb and MURF2 mRNAs apparently arise due to the reduced area of editing required. It is not known why different mRNAs require editing to different extents. One possibility is that the PF cell relies heavily on CYb and MURF2, and that evolution of these mRNAs has been constrained to a small region thereby allowing production of relatively large amounts of fully edited mRNAs. Investigation of IPSs in CYb and MURF2 mRNA editing revealed intrinsic pauses primarily at or very near sites of gRNA annealing/exchange, suggesting that barriers to editing of these mRNAs occur primarily due to effects on gRNA/mRNA annealing. This contrasts with the IPSs of pan-edited mRNAs, which are present throughout gRNA-defined blocks and thereby indicate barriers to gRNA utilization (9). Minimally edited mRNAs, on the other hand, have fewer intrinsic barriers to gRNA utilization, perhaps due to decreased intra-mRNA and mRNA/gRNA structures, which are thought to pose obstacles for editing of pan-edited mRNAs. Reduced structure could be the result of an unknown protein constraining the region of editing and may explain why TbRGG2 has little effect on editing of minimally edited mRNAs ((13) and data herein). TbRGG2 modulates mRNA/gRNA interactions during editing progression on pan-edited mRNAs (9,12), and if such interactions are being constrained by some other factor in minimally edited mRNAs, then TbRGG2 would have little impact on the editing of those mRNAs. Thus, while overall editing efficiency is comparable between minimally edited and pan-edited mRNAs, the mechanisms that constrain editing efficiency appear to differ between the two classes of edited mRNAs.

Data presented here contribute to our understanding of the junction regions that are characteristic of most partially edited mRNAs. The origins and functions of junctions are a subject of debate as discussed in detail in a recent review (31). Our data provide evidence for three mechanisms of junction formation: misalignment between the mRNA and canonical gRNA, inefficient gRNA anchoring and incorrect gRNA utilization. With regard to misalignment, it is easy to envision that intra-mRNA folding within a gRNA-directed region could lead to erroneous gRNA annealing upstream or downstream of its canonical site, thereby leading to modifications directed by the gRNA being shifted several ESs, forming a junction. Evidence of this phenomenon was observed in regions of 8 U insertions following CYb Editing Stop Site 558 (Figure 2B) and 9 U insertions following MURF2 Editing Stop Site 446 (Figure 3C). In CYb mRNA, we also noted that inefficient usage of gCYb-2 appeared to lead to significant intrinsic pauses in canonical editing at the end of the region directed by the first gRNA. mRNAs with junction length zero were prominent at Editing Stop Site 569 (Figure 2C). The abundance of these mRNAs indicates accumulation of mRNAs that have been correctly edited throughout the length of the first gRNA but for which editing directed by the second gRNA has not commenced, suggesting that gRNA exchange is a significant barrier to progression. The other most abundant mRNAs with Editing Stop Site 569 exhibit incorrect editing at ES570 and ES571. Thus, even when gCYb-2 is being utilized certain regions or ESs on the mRNA, such as ES570, may be challenging for the machinery to properly edit, leading to junction formation. Finally, annealing of an incorrect or alternative gRNA can promote the production of non-canonical modifications, which are detected as junctions. Analysis of the gRNA transcriptome database established by Koslowsky, *et al.* (11) revealed novel gRNAs that could direct editing patterns observed in long junctions in both CYb and MURF2 mRNAs (Figures 2A and 3B). Remarkably, we also detected an abundant MURF2 mRNA whose editing appeared to be directed in part by a gRNA (alt-MURF2-gRNA2) that was previously reported to direct editing of the CR4 mRNA (11). This finding reveals that gRNA selection is error prone even in wild-type cells, provided that the anchor regions between two mRNAs are similar enough for a gRNA to anneal to either mRNA.

Junctions have been proposed to be dead end products, regions of active editing that undergo multiple rounds of remodeling, or alternative sequences that ultimately diversity the proteome (9,31–36). *In silico* analysis of an abundant class of partially edited CYb mRNAs whose editing is apparently directed by an alternative gRNA (Figure 2A) indicated that these junction containing mRNAs are either dead end products or that they would require remodeling to generate translatable mRNAs. Evidence of junction remodeling came from analysis of MURF2 mRNAs with Editing Stop Site 441, whose 3′ editing could be directed in part by alt-MURF2-gRNA1. Sequences 2, 7 and 9 in Figure 3A are consistent with edited sequence initially directed by alt-MURF2-gRNA1 at ES447 and ES448 undergoing subsequent remodification by the canonical gMURF2. Moreover, we previously reported data indicating that junction formation is, at least in some cases, essential for editing progression, thereby further supporting a role for junctions as regions of active editing (9). Finally, a subset of partially edited MURF2 mRNAs reported here with junctions at ES447/448 and extensive canonical 5′ sequence could be *in silico* translated into proteins with limited, conserved amino acid changes compared to canonical MURF2. However, the extent of potential proteome diversification observed here is modest compared to that suggested by some previous studies for other edited mRNAs (40,42). Overall, junction regions appear to be ubiquitous, with multiple methods of formation and potential functions. Future analysis of partially edited mRNA populations using high-throughput methods is likely to provide continued insights into this important aspect of U insertion/deletion RNA editing.

Using TREAT, we revisited the functions of editing accessory factors MRP1/2 and RBP16. RBP16 primarily affects CYb editing at the level of initiation. In contrast, MRP1/2 depletion leads to a 75% reduction in total CYb mRNA, and almost complete loss of partially and fully edited CYb mRNAs. Because the level of total CYb mRNA remaining after MRP1/2 knockdown (25%; Figure 4B) is almost identical to the percent of pre-edited mRNAs in uninduced cells (27%; Figure 4A), these data strongly suggest that MRP1/2 maintains edited CYb mRNA by stabilizing mRNAs that have entered the editing pathway. Neither factor influences the 3′ to 5′ progression of editing along CYb mRNA. With regard to MURF2 mRNA, MRP1/2 depletion again leads to a dramatic reduction in total mRNA levels and in fully edited mRNA. However, in contrast to CYb mRNA, the proportion of partially edited MURF2 mRNA is not decreased, and in fact is significantly increased when MRP1/2 is knocked down. RBP16 knockdown also leads to a decrease in fully edited mRNA, and corresponding increases in pre-edited and partially edited mRNA, although total mRNA levels are essentially unchanged. The observed increases in pre-edited mRNA indicate that both factors impact MURF2 editing initiation. TREAT analysis also revealed that both RBP16 and MRP1/2 affect editing progression on MURF2 mRNA. These factors are likely involved in mRNA/gRNA annealing in MURF2 editing considering the positions of EPSs found in the sequences as well as their known biochemical activities (18,21–23).

Accumulation of edited CYb mRNA is strictly developmentally regulated, and our findings regarding MRP1/2 and RBP16 suggest that these factors could be involved in this regulation. PF *T. brucei* contain abundant edited CYb mRNA, whereas edited CYb mRNA is almost undetectable in bloodstream form (BF) cells (30,36,43). Knockdown of MRP1/2 resulted in a CYb mRNA population that is almost entirely pre-edited, similar to the state of the CYb mRNA population in BF. Although MRP1/2 is abundant in BF, the ability of MRP1/2 to stabilize edited CYb mRNA may be compromised in this life cycle stage. For example, MRP1/2 binding sites on CYb mRNA may be occluded by binding of a BF-specific protein that successfully outcompetes MRP1/2, or MRP1/2 could be differentially posttranslationally modified in BF. MRP1/2 is not essential for growth of BF (45), consistent with the dispensability of edited CYb mRNA in this stage. Likewise, if RBP16 is needed to promote entry of pre-edited CYb mRNA into the editing pathway, life cycle-specific modifications or competing CYb mRNA binding proteins could negatively affect its function in BF. Interestingly, RBP16 undergoes arginine methylation in PF *T. brucei* and this modification promotes its association with mRNAs, including pre-edited and edited CYb (46–49). The methylation status of RBP16 in BF is unknown. MRP1 is multiply phosphorylated, and serine phosphorylation at S198 is over ten-fold increased in PF compared to BF (50). Thus, it will be of interest in the future to determine these modifications of RBP16 and MRP1/2 affect their interactions with CYb mRNA in a life cycle stage-specific manner.

High-throughput sequencing studies here show that CYb mRNA editing is not significantly affected by TbRGG2 depletion, a finding consistent with previous results (13). Instead, the impact of TbRGG2 on CYb mRNA editing is likely to entail a number of small effects that we observe as EPSs (Figure 7), but which do not together constitute a significant effect on the abundances of pre-edited or fully edited mRNA. The EPSs we observe in CYb mRNA upon TbRGG2 knockdown are phenotypically the same as what we see for pan-edited mRNAs in that they occur in the middle of gRNA-defined blocks (9). The effect of TbRGG2 on MURF2 mRNA editing, while stronger than for CYb, again entails a modest effect on editing progression. Upon TbRGG2 depletion, we also see a significant increase in sequences with shorter junctions (Supplementary Figure S4), a finding that consistent with published TbRGG2 function (9). Together, analysis of TbRGG2 knockdown cells show that, although the effect of TbRGG2 is substantially decreased with respect to minimally edited mRNAs, this factor has a similar qualitative function in the editing of both pan-edited and minimally edited mRNAs.

GAP1/2 has a well-established function, being essential for stabilization of the entire mitochondrial gRNA population (15,16). Consequently, our previous studies of pan-edited mRNAs have shown effects of GAP1/2 knockdown primarily on editing initiation and EPSs at the ends of gRNAs (9). The occurrence of EPSs within gRNA blocks for both CYb and MURF2 that we demonstrate here reveals a possible new role for GAP1/2 in facilitating progression of editing through minimally edited mRNAs. GAP1/2 reportedly forms complexes apart from fully assembled RESC with other editing factors and RNA binding proteins such as REH2, MRB7260 and TbRGG3 (10,38,39,51). One or more of these complexes may carry out additional functions of GAP1/2. Thus, it will be of future interest to examine editing defects by high-throughput sequencing in cells depleted of these GAP1-associated factors. Such studies may reveal functions of distinct GAP1/2-containing complexes in gRNA utilization and editing progression.

## Supplementary Material

gkz012_Supplemental_Files
