# Supplementary material for: Intrinsic and regulated properties of minimally edited trypanosome mRNAs
Source: Nucleic Acids Res. 2019 Jan 30;47(7):3640–57. doi: 10.1093/nar/gkz012 (PMC6468165; doi:10.1093/nar/gkz012)
Supplement: gkz012_Supplemental_Files [file gkz012_supplemental_files.zip › Supplementary Data.pdf]

Figure S1

A

RNAi Cell Line	Rep1 - Tet	Rep1 + Tet	Rep2 - Tet	Rep2 + Tet
MRP1/2	1.2×10^7	9.2×10^6	7.4×10^6	5.2×10^6
RBP16	1.1×10^7	4.7×10^6	1.1×10^7	5.0×10^6
TbRGG2	4.6×10^6	3.0×10^6	8.0×10^6	9.4×10^6
GAP1	5.0×10^6	4.7×10^6	1.0×10^7	9.7×10^6

B

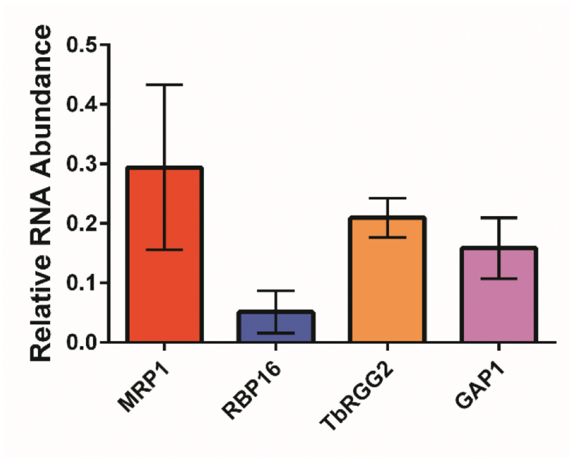

C

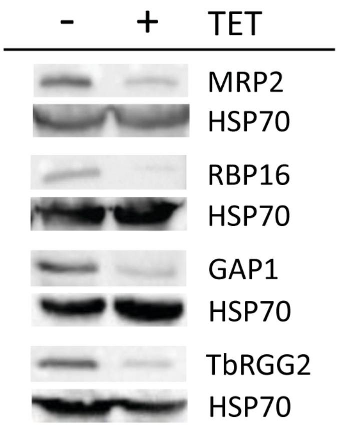

Figure S2

Uninduced

```
                                                                                                439
5'                                                                                               ▼            3'
AAUCUAUAAUG-A----A--A-G---G-----G---G----AUUUUA----A--G----A---UUG-----G-CUUUG--AUUG----A-----------GUCGUGUUUUUGA  Pre-edited
AAUCUAUAAUG-AuuuuA--AuGuuuG-----G-uuGuuuuA****A-uuuA--GuuuuAuuuUUG----uG-CUUUG--AUUG---uA-----------GUCGUGUUUUUGA  Edited
         : ||||||  |::|:::     : ||::|:||    | ||||  :|||||||||||    || |||:   ||
      7UC3UU-UAAAAU--UGUAGGU-----U-AAUGAGAU----U-AAAU--UAAAAUAAAAAC----AC-GAAGA--UA  gMURF2
                                                                                                                   ESS   JL   Avg ReNorm
AAUCUAUAAUG-AuuuuA--AuGuuuG-----G-uuGuuuuA****A-uuuA--GuuuuAuuuUUG----uG-CuuuG--AuuG---uAu----------GUCGUGUUUUUGA  439   19   14.7
AAUCUAUAAUG-A----A--A-G--uG-----GuuuG----A****AuuuuAuuG---uA---*UGuuuuuG-C--uGuuA--G----Au----------GUCGUGUUUUUGA  439   16   13.1
AAUCUAUAAUG-A----A--A-G--uG-----GuuuG----A****AuuuuAuuG---uA---*UGuuuuuG-C--uGuuA--G----Auu---------GUCGUGUUUUUGA  439   16    6.6
AAUCUAUAAUG-A----A--A-G--uG-----GuuuG----A***UA---uAuuG---uA---*UGuuuuuG-C--uGuuA--G----Au----------GUCGUGUUUUUGA  439   16    6.4
AAUCUAUAAUG-A----A--A-G---G-----G---G----AUUUUAuuuuAuuG---uA---*UGuuuuuG-C--uGuuA--G----Au----------GUCGUGUUUUUGA  439   12    6.2
AAUCUAUAAUG-A----A--A-G---G-----G---G----A***UA----A--G----A--uUUG-----GuC---G-uA--GuuuuAuuuuuuuuuuuGUCGUGUUUUUGA  439   12    4.4
AAUCUAUAAUG-A----A--A-G---G-----G---G----AUUUUAuuuuAuuG---uA---*UGuuuuuG-C--uGuuA--G----Au----------GUCGUGUUUUUGA  439   11    4.2
AAUCUAUAAUG-AuuuuA--AuGuuuG-----G-uuG----A****AuuuuAuuG---uA---*UGuuuuuG-C--uGuuA--G----Au----------GUCGUGUUUUUGA  439   19    3.9
AAUCUAUAAUG-A----A--A-G---G----uG-uuG----A****AuuuuAuuG---uA---*UGuuuuuG-C--uGuuA--G----Au----------GUCGUGUUUUUGA  439   15    3.7
```

MRP1/2 KD

```
                                                                                                439
5'                                                                                               ▼          3'
AAUCUAUAAUG-A----A--A-G---G-----G---G----AUUUUA----A--G----A---UUG-----G-CUUUG--AUUG----A-----------GUCGUGUUUUUGA  Pre-edited
AAUCUAUAAUG-AuuuuA--AuGuuuG-----G-uuGuuuuA****A-uuuA--GuuuuAuuuUUG----uG-CUUUG--AUUG---uA-----------GUCGUGUUUUUGA  Edited
         : ||||||  |::|:::     : ||::|:||    | ||||  :|||||||||||    || |||:   ||
      7UC3UU-UAAAAU--UGUAGGU-----U-AAUGAGAU----U-AAAU--UAAAAUAAAAAC----AC-GAAGA--UA  gMURF2
                                                                                                                   ESS   JL   Avg ReNorm
AAUCUAUAAUG-AuuuuA--AuGuuuG-----G-uuGuuuuA****A-uuuA--GuuuuAuuuUUG----uG-CuuuG--AuuG---uAu----------GUCGUGUUUUUGA  439   19   33.7
AAUCUAUAAUG-A----A--A-G---G----uG-uuG----A****AuuuuAuuG---uA---*UGuuuuuG-C--uGuuA--G----Au----------GUCGUGUUUUUGA  439   15   32.4
AAUCUAUAAUG-A----A--A-G---G-----G---G----AUUUUAuuuuAuuG---uA---*UGuuuuuG-C--uGuuA--G----Au----------GUCGUGUUUUUGA  439   12   32.0
AAUCUAUAAUG-A----A--A-G---G-----G---G----AUUUUAuuuuAuuG---uA---*UGuuuuuG-C--uGuuA--G----Au----------GUCGUGUUUUUGA  439   11   26.2
AAUCUAUAAUG-A----A--A-G--uG-----GuuuG----A****AuuuuAuuG---uA---*UGuuuuuG-C--uGuuA--G----Au----------GUCGUGUUUUUGA  439   16   21.2
AAUCUAUAAUG-A----A--A-G---G-----G---G----AUUUUAuuuuAuuG---uA---*UGuuuuuG-C--uGuuA--G----Auu---------GUCGUGUUUUUGA  439   12   20.0
AAUCUAUAAUG-A----A--A-G---G----uG-uuG----A****AuuuuAuuG---uA---*UGuuuuuG-C--uGuuA--G----Auu---------GUCGUGUUUUUGA  439   15   19.7
AAUCUAUAAUG-A--uuAuuAuG---G----uG-uuG----A****AuuuuAuuG---uA---*UGuuuuuG-C--uGuuA--G----Au----------GUCGUGUUUUUGA  439   19   19.4
AAUCUAUAAUG-A----A--A-G---G-----G---G----AUUUUAuuuuAuuG---uA---*UGuuuuuG-C--uGuuA--G----Auu---------GUCGUGUUUUUGA  439   11   15.4
```

RBP16 KD

```
                                                                                                 439
5'                                                                                                ▼          3'
AAUCUAUAAUG-A----A--A--G---G-----G---G----AUUUUA----A--G----A---UUG-----G-CUUUG--AUUG----A-----------GUCGUGUUUUUGA  Pre-edited
AAUCUAUAAUG-AuuuuA--A-uGuuuG-----G-uuGuuuuA****A-uuuA--GuuuuAuuuUUG----uG-CUUUG--AUUG---uA-----------GUCGUGUUUUUGA  Edited
         : ||||||  | ::|:::     : ||::|:||    | ||||  :|||||||||||    || |||:   ||
      7UC3UU-UAAAAU--U-GUAGGU-----U-AAUGAGAU----U-AAAU--UAAAAUAAAAAC----AC-GAAGA--UA  gMURF2
                                                                                                                    ESS   JL   Avg ReNorm
AAUCUAUAAUG-AuuuuA--A-uGuuuG-----G-uuGuuuuA****A-uuuA--GuuuuAuuuUUG----uG-CuuuG--AuuG---uAu----------GUCGUGUUUUUGA  439   19   21.9
AAUCUAUAAUG-A----A--A--G--uG-----GuuuG----A****AuuuuAuuG---uA---*UGuuuuuG-C--uGuuA--G----Au----------GUCGUGUUUUUGA  439   16   18.9
AAUCUAUAAUG-A----A--A--G---G-----G---G----A***UA----A--G----A--uUUG-----GuC---G-uA--GuuuuAuuuuuuuuuuuGUCGUGUUUUUGA  439   12   18.8
AAUCUAUAAUG-A----A--AuuG-uuGuuuuuG-uuG--uuA***UA---uA-uG----A---**GuuuuuGuC---GuuA--G----Au----------GUCGUGUUUUUGA  439   17   18.2
AAUCUAUAAUG-A----A--A--G---G-----G---G----AUUUUAuuuuAuuG---uA---*UGuuuuuG-C--uGuuA--G----Au----------GUCGUGUUUUUGA  439   12   14.7
AAUCUAUAAUG-A----A--A--G--uG-----GuuuG----A***UA---uAuuG---uA---*UGuuuuuG-C--uGuuA--G----Au----------GUCGUGUUUUUGA  439   16   12.3
AAUCUAUAAUG-AuuuuA--A-uGuuuG-----G-uuG----A****AuuuuAuuG---uA---*UGuuuuuG-C--uGuuA--G----Au----------GUCGUGUUUUUGA  439   19   11.2
AAUCUAUAAUG-AuuuuA--A--G--uG-----GuuuG----A****AuuuuAuuG---uA---*UGuuuuuG-C--uGuuA--G----Au----------GUCGUGUUUUUGA  439   19   11.0
AAUCUAUAAUG-AuuuuA--A-uGuuuG-----G-uuGuuuuA****AuuuuAuuG---uA---*UGuuuuuG-C--uGuuA--G----Au----------GUCGUGUUUUUGA  439   19   10.6
```

Figure S3

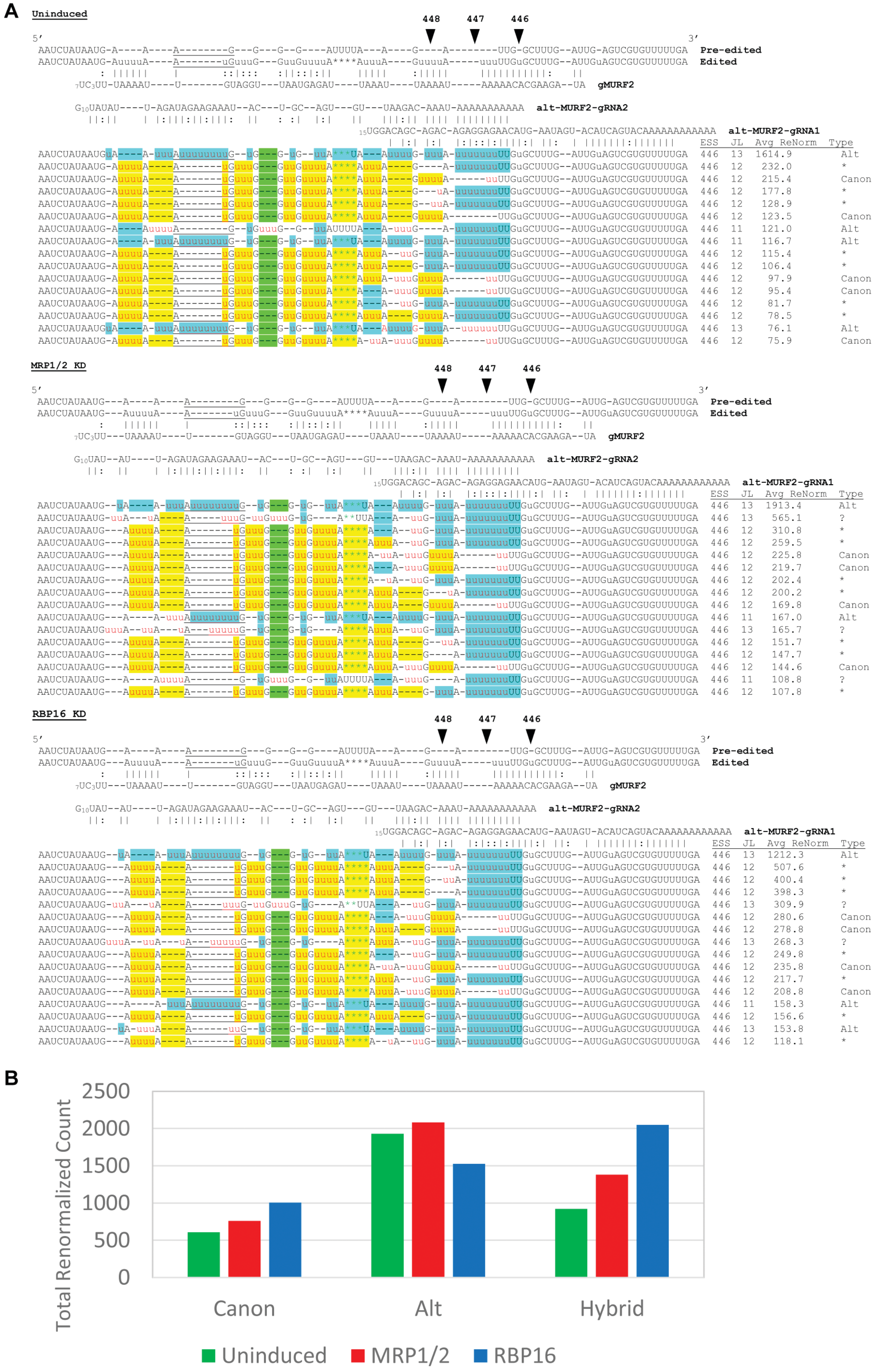
A
Uninduced
MRP1/2 KD
RBP16 KD
448
447
446
5′
3′
Pre-edited
Edited
gMURF2
alt-MURF2-gRNA2
alt-MURF2-gRNA1
ESS
JL
Avg ReNorm
Type
Alt
Canon
B
Total Renormalized Count
2500
2000
1500
1000
500
0
Canon
Alt
Hybrid
Uninduced
MRP1/2
RBP16

Figure S4

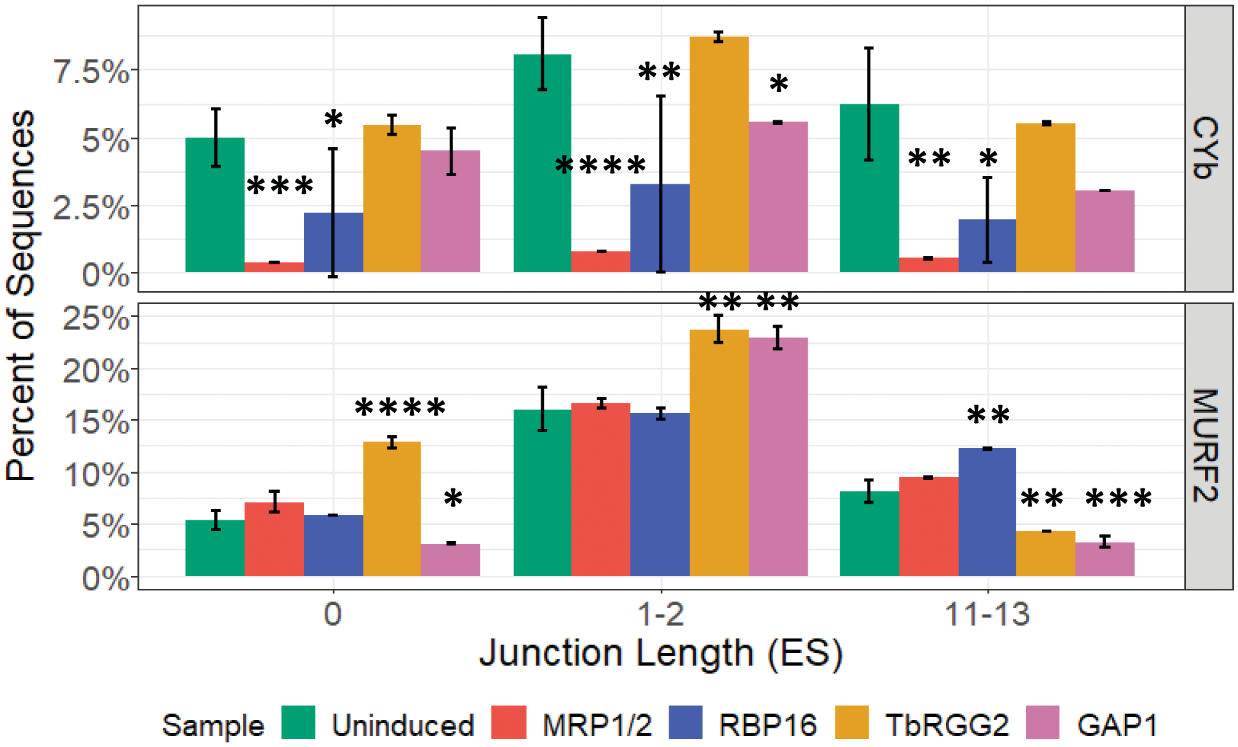

Percent of Sequences
7.5%
5%
2.5%
0%
25%
20%
15%
10%
5%
0%
CYb
MURF2
0
1-2
11-13
Junction Length (ES)
Sample
Uninduced
MRP1/2
RBP16
TbRGG2
GAP1

Figure S5

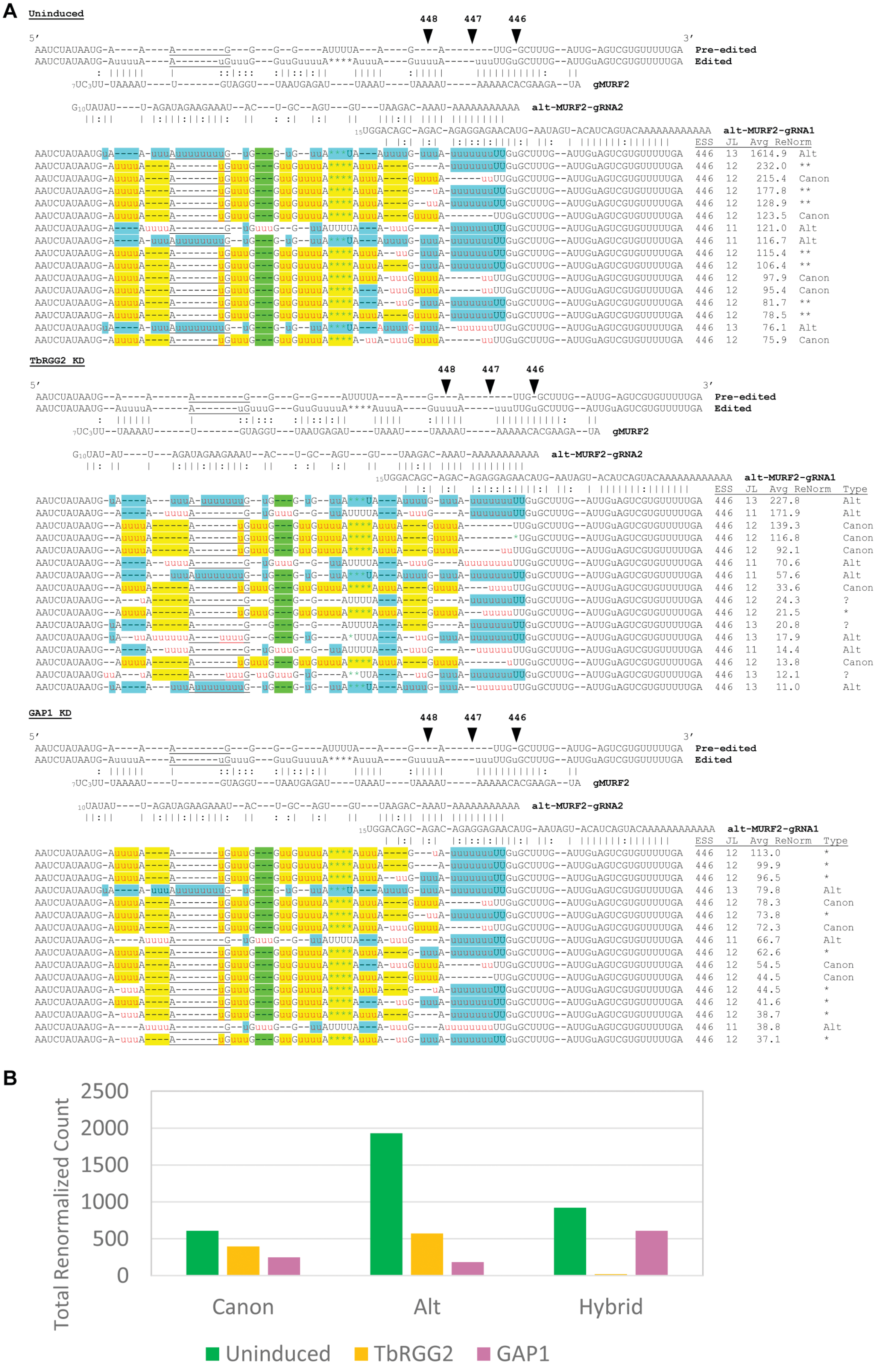

A
Uninduced
448
447
446
5'
3'
AAUCUAUAAUG-A----A----A--------G---G---G--G----AUUUUA---A----G----A--------UUG-GCUUUG--AUUG-AGUCGUGUUUUUGA
Pre-edited
Edited
gMURF2
alt-MURF2-gRNA2
alt-MURF2-gRNA1
ESS
JL
Avg ReNorm
Type
Alt
Canon
TbRGG2 KD
GAP1 KD
B
Total Renormalized Count
2500
2000
1500
1000
500
0
Canon
Alt
Hybrid
Uninduced
TbRGG2
GAP1

Fig. S1. RNAi cell lines analyzed in this study. (A) The concentration (cells/mL) of all uninduced (-Tet) and induced (+Tet) RNAi cell lines at time of RNA collection (day 3 post-induction). (B) Relative abundances (induced/uninduced) of target RNAs analyzed by qRT-PCR at the time of RNA collection (n=6 replicates). (C) Western blot analysis of target RNAs in uninduced (-Tet) and induced (+Tet) RNAi cell lines at time of RNA collection. HSP70 is a loading control.

Fig. S2. Sequence analysis of the most abundant junctions arising at MURF2 ESS439 in Uninduced, MRP1/2, and RBP16 cell lines. For each cell type the renormalized counts of junction sequences were averaged across all uninduced cell lines in this study (n=8) and induced replicates for each knockdown (n=2 for each cell line). The nine most abundant junctions arising in each cell type were then aligned according to their non-U sequence. MURF2 pre-edited and fully edited sequence, along with the previously identified gRNA-MURF2, are shown above. Editing sites that match the canonical sequence are highlighted in yellow. Lower case u's are those inserted by editing, U indicates encoded uridines, and * indicates encoded uridines deleted by editing.

Fig. S3. Sequence analysis of the most abundant junctions arising at MURF2 ESS446 in Uninduced, MRP1/2, and RBP16 cell lines. (A) For each cell type, the renormalized counts of junction sequences were averaged across all uninduced cell lines used in this study (n=8) and induced replicates (n=2 for each knockdown). To evaluate gRNA usage, the 16 most abundant junctions of lengths 11 editing sites or longer were aligned according to their non-U sequence. MURF2 pre-edited and fully edited sequence, along with the previously identified gMURF2, are shown above. Editing sites which that the canonical sequence are highlighted in yellow, while editing sites matching modifications made by alt-MURF2-gRNA2 are highlighted in blue. Green highlighting represents editing sites whose editing is are correctly edited by either gMURF2 or alt-MURF2-gRNA. Sequences were classified according to their complementarity to the canonical gRNA-MURF2 (Canon), alt-MURF2-gRNA2 (Alt), as hybrid junctions (*), or as undeterminable (?). (B) Quantification of junction classes shown in (A). Canon, canonical as specified by gMURF2; Alt, alternative as specified by alt-MURF2-gRNA2; Hybrid; containing 9 U's at ES447, but canonical sequences both 5' and 3' of this site.

Fig. S4. Overall changes in junction lengths. The percentage of normalized sequences (excluding pre-edited) with small (0-2) and long (11-13) junction lengths were calculated across (n=8) uninduced and (n=2) induced replicates for each knockdown. Error bars represent one standard deviation. * $P < 0.05$; ** $P < 0.01$; *** $P < 0.001$; **** $P < 0.0001$ (Student's T-test).

Fig. S5. Sequence analysis of the most abundant junctions arising at ESS446 in Uninduced, TbRGG2, and GAP1 cell lines. (A) For each cell type, the renormalized counts of partially edited sequences were averaged (Avg ReNorm) across all uninduced cell lines used in this study (n=8) and induced knockdown cells (n=2 for each cell line). To evaluate gRNA usage, the most abundant 16 junctions comprised of 11 editing sites or longer were aligned according to their non-U sequence. MURF2 pre-edited and fully edited sequence, along with the gMURF2, alt-MURF2-gRNA1, and alt-MURF2-gRNA2 are shown above. Editing sites that match the canonical sequence are highlighted in yellow while editing sites matching modifications made by alt-MURF2-gRNA2 are highlighted in blue. Green highlighting represents editing sites that are correctly edited according to either gRNA. Sequence types (Type) were classified according to

complimentary to the canonical gRNA-MURF2 (Canon), alt-MURF2-gRNA2 (Alt), as hybrid junctions (*), or as indeterminable (?). Lower case u's are those inserted by editing, U indicates encoded uridines, and * indicates encoded uridines deleted by editing. (B) Quantification of junction classes shown in (A).
